# Evidence for SH2 Domain-Containing 5′-Inositol Phosphatase-2 (SHIP2) Contributing to a Lymphatic Dysfunction

**DOI:** 10.1371/journal.pone.0112548

**Published:** 2014-11-10

**Authors:** Germaine D. Agollah, Manuel L. Gonzalez-Garay, John C. Rasmussen, I-Chih Tan, Melissa B. Aldrich, Chinmay Darne, Caroline E. Fife, Renie Guilliod, Erik A. Maus, Philip D. King, Eva M. Sevick-Muraca

**Affiliations:** 1 Center for Molecular Imaging, The Brown Foundation Institute of Molecular Medicine, The University of Texas Health Science Center, Houston, Texas, United States of America; 2 The University of Texas Graduate School of Biomedical Sciences at Houston, The University of Texas MD Anderson Cancer Center, Houston, Texas, United States of America; 3 Division of Genomics and Bioinformatics, Center for Molecular Imaging, The Brown Foundation Institute of Molecular Medicine, Houston, Texas, United States of America; 4 Memorial Hermann Hospital Center for Lymphedema Management, Houston, Texas, United States of America; 5 Department of Microbiology and Immunology, University of Michigan Medical School, Ann Arbor, Michigan, United States of America; McGill University, Canada

## Abstract

The lymphatic vasculature plays a critical role in a number of disease conditions of increasing prevalence, such as autoimmune disorders, obesity, blood vascular diseases, and cancer metastases. Yet, unlike the blood vasculature, the tools available to interrogate the molecular basis of lymphatic dysfunction/disease have been lacking. More recently, investigators have reported that dysregulation of the PI3K pathway is involved in syndromic human diseases that involve abnormal lymphatic vasculatures, but there have been few compelling results that show the direct association of this molecular pathway with lymphatic dysfunction in humans. Using near-infrared fluorescence lymphatic imaging (NIRFLI) to phenotype and next generation sequencing (NGS) for unbiased genetic discovery in a family with non-syndromic lymphatic disease, we discovered a rare, novel mutation in *INPPL1* that encodes the protein SHIP2, which is a negative regulator of the PI3K pathway, to be associated with lymphatic dysfunction in the family. *In vitro* interrogation shows that SHIP2 is directly associated with impairment of normal lymphatic endothelial cell (LEC) behavior and that SHIP2 associates with receptors that are associated in lymphedema, implicating its direct involvement in the lymphatic vasculature.

## Introduction

Pathophysiological processes that exhibit loss of fluid homeostasis, disrupted lipid absorption and transport, and compromised immune surveillance/response generally have some abnormal involvement of the lymphatic circulatory system [1]. Indeed, many prevalent diseases and conditions (including cancer metastasis, diabetes, asthma, obesity, and autoimmune disorders) are thought to be impacted by aberrant lymphangiogenesis or lymphatic dysfunction [2]–[4]. Genetic studies of rare congenital lymphatic disorders provide an opportunity to identify key molecular mechanisms that may be contributory to more common conditions and disorders. From candidate gene studies, causal genes including *FOXC2, FLT4, SOX18, CCBE1, GJC2, GATA2, KIF11, RASA1*, and *VEGFC* have thus far been associated with lymphatic malformations and congenital lymphedema that generally presents with variable penetrance and expressivity, often late in life [5]–[14]. Yet the majority of patients diagnosed with lymphatic abnormalities do not possess mutations in these genes.

One confounding limitation to the study of human lymphatic disorders arises from the lack of diagnostic methods to image the lymphatic vasculature. As a result, diagnoses of rare, lymphatic disorders are not generally made on the basis of abnormal lymphatic architecture or dysfunction, but rather on the late-stage sequelae of fluid imbalances, such as irresolvable edema, chylothorax, or chylous ascites. Edematous symptoms typically appear at birth (termed congenital lymphedema), at puberty (termed *praecox* lymphedema), or late in life, usually following a minor challenge to the immune system (termed *en tarda* lymphedema). As a consequence of the variable expressivity, the inaccuracy of phenotyping based upon overt symptoms can limit the association with a found genotype and therefore impede discovery of gene variants involved in lymphatic (dys)function.

Herein, in an effort to discover novel genetic mutations that contribute to lymphatic disease, we used next generation sequencing (NGS) for unbiased gene search and near-infrared fluorescence lymphatic imaging (NIRFLI) to phenotype family members and discovered that a mutation in the inositol polyphosphate phosphatase-like 1 (*INPPL1*) gene that encodes SH2-domain containing 5′-inositol phosphatase-2 (SHIP2) may be associated with lymphatic abnormalities in a nucleus family of varied expressivity of lymphedema. When the identified SHIP2 mutation was combined with a damaging mutation in hepatic growth factor (HGF), the ligand for the receptor tyrosine kinase (RTK) cMET, family members suffered more severe forms of lymphedema than those who possessed the SHIP2 mutation alone, while no lymphatic abnormalities were associated with the HGF mutation alone. WES analyses of additional family members reveal the same SHIP2 mutation in members with various diagnoses of lymphedema.

SHIP2 is a phosphatidylinositol (3,4,5) triphosphate (PIP3) 5′-phosphatase that negatively controls PIP3 levels in cells, thereby inhibiting the phosphatidylinositol-3-kinase (PI3K)/AKT signaling pathway. Recently mutations within the PI3K/AKT pathway have been implicated in various lymphatic malformations and syndromic conditions that have a lymphatic involvement. CLOVES syndrome (OMIM: 612918) and Klippel-Trenaunay-Weber syndrome (KTWS, OMIM: 149000) are caused by gain-of-function mutations in *PI3KCA*, which encodes p110α, a catalytic subunit of PI3K, while *AKT1* mutations have been identified in Proteus syndrome (OMIM: 176920) [15]–[17]. In addition, in mice, targeted mutations of *Pik3r1*, *Pi3kca* and *Akt1* genes result in lymphatic abnormalities including lymphangiectasia, defective lymphatic sprouting and maturation, lymphatic vessel hypoplasia, and chylous ascites [18]–[20]. SHIP2 is known to interact physically with RTKs, focal adhesion proteins, scaffold proteins, protein phosphatases, and cytoskeletal proteins to regulate cell proliferation, adhesion, migration, survival, and receptor internalization [21]–[24]. Therefore, given the evidence that PI3K/AKT pathway plays a role in lymphatic malformations, one may hypothesize that SHIP2, a negative regulator of this pathway, could be involved in lymphatic disorders. Through *in vitro* siRNA studies, we show that SHIP2 is required for the proliferation, adhesion, migration, tubulogenesis, and survival of human lymphatic endothelial cells (LEC). When the mutant form of SHIP2 discovered in our human studies was overexpressed in immortalized LEC, the cellular phenotypes were found to differ as compared to those overexpressing wild type (WT) SHIP2. The combined results suggest that SHIP2 is a potential modulator of lymphatic function and that SHIP2 mutations can contribute to varied expressivity and penetrance of lymphatic disorders.

## Materials and Methods

### Human Lymphatic Imaging

The lymphatic phenotype of each subject was determined using near-infrared fluorescence lymphatic imaging (NIRFLI). As part of an ongoing, University of Texas Health Science Center institutional review board (IRB) and FDA-approved study of lymphatic disorders conducted under Food and Drug Administration IND# 102,827 clinical study and funded by the National Heart Lung and Blood Institute of the National Institutes of Health (Clinical Trials No. NCT00833599: “Imaging lymphatic function in normal subjects and in persons with lymphatic disorders” www.clinicaltrials.gov), subjects were imaged following intradermal injection of indocyanine green (ICG) and DNA collection was performed. All clinical investigations were conducted according to Declaration of Helsinki principles. Written informed consent was obtained from all participants prior to NIRFL imaging.

**Figure 1:**
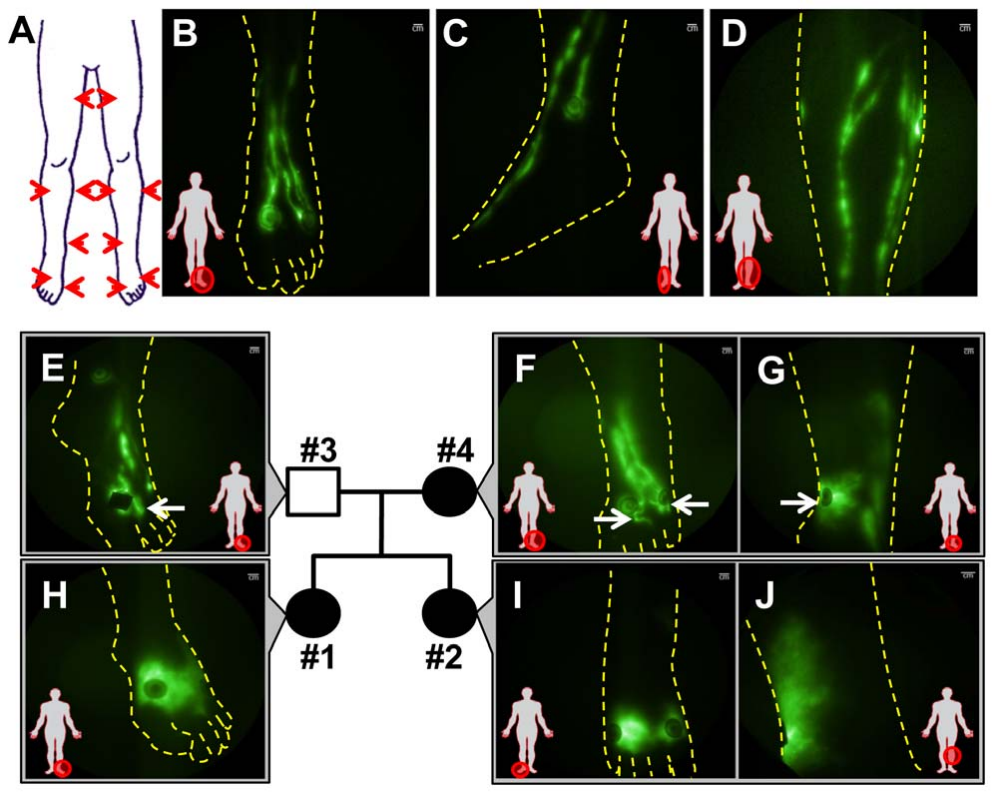
Aberrant lymphatic phenotype of familial lymphedema imaged with NIRFLI. **(A)** Schematic illustrating the typical location of the intradermal injections administered in each subject. NIRF images of normal lymphatic vasculature in the (**B**) foot, (**C**) medial ankle, and (**D**) shin as outlined with the dotted lines. Lymphatic vasculature observed in the nucleus family. The father (Subject #3; unaffected) had well-defined lymphatics in both legs except a few lymphatic capillaries radiated from injection sites on the left foot (arrow) (**E**). The mother (Subject #4; *en tarda* in left leg and acquired lymphedema in right leg) had tortuous, lymphatic capillaries (arrows) radiating from injection sites in the left foot (**F**) and an area of dermal backflow (arrow) in the medial ankle (**G**). The older daughter (Subject #1; left unilateral lymphedema *praecox*) had dermal backflow in the left foot (**H**). The younger daughter (Subject #2; bilateral congenital lymphedema) had extensive dermal backflow in both limbs as shown in the right foot (**I**) and left lateral calf (**J**). Injection sites are covered with round adhesive bandages and/or black vinyl tape to avoid camera oversaturation. Scale bar  = 1 cm.

Each subject received a total of 12 intradermal injections of 25 µg of ICG in 0.1 mL saline, with a total dose of 300 µg, for uptake into the peripheral lymphatics. Injection sites were consistent between subjects and each limb received two injections on the dorsum of the foot, one injection on the medial ankle, one injection each on the lateral and medial calf, and one injection on the anterior thigh (see **Fig 1A**). Immediately following ICG administration and as described previously 25 each leg was illuminated by the diffuse output of a 785 nm laser diode, and images of the resultant fluorescent signal emanating from the peripheral lymphatics were optically filtered and acquired using a custom intensified charge coupled device (ICCD) camera. Exposure times of 200 ms permitted the acquisition of movies of lymphatic contractile function. Vital signs were measured for two hours following injection, and a follow-up phone call was made at 24 hours. Blood was obtained from each subject. No adverse events were reported. Images were analyzed to assess lymphatic uptake at the injection site and the presence of abnormal lymphatic architecture such as dense networks of lymphatic capillaries with extravascular fluorescence frequently known as dermal backflow, lymphatic capillaries radiating from the intradermal injection sites, as well as tortuous and/or dilated lymphatics. The brightness and contrast levels of the NIRFLI images presented herein were adjusted to increase the overall dynamic range observable in the image (i.e. to enable the visualization of both the dim and the bright signals within the lymphatics). The NIRF images are presented in green pseudo-color.

### Whole Exome Sequencing (WES)

DNA was extracted from blood using Paxgene Blood DNA Kit (PreAnalytix, Switzerland) according to the vendor's instructions. 2 µg of genomic DNA were submitted to Axeq Technologies for human exome capture sequencing using TrueSeq 62-Mb target enrichment (Illumina, San Diego, CA).

### Genetic Sequencing Analysis

Each pair of fastq files was aligned to human genome (hg19) using Novoalign (Novocraft Technologies; www.novocraft.com), keeping parameters at the default settings, as recommended by Novocraft Technologies. Novoalign, SAMtools (http://samtools.sourceforge.net) was used to sort the SAM files, create BAM files, and generate their index files. Picard (SourceForge; http://picard.sourceforge.net) was used to remove all of the PCR duplicates from the BAM files. For local realignments, base quality recalibration, and variant calling, we used Genome Analysis Toolkit (GATK) Version 2.2. Finally, for variant annotation, we used SnpEff (http://snpeff.sourceforge.net/), variant tools and ANNOVAR using multiple databases from UCSC Genome bioinformatics. Functional effects of each non-synonymous coding variant were evaluated using three different functional prediction algorithms: (1) Polyphen 2.0 Prediction of functional effects of human nsSNPs (http://genetics.bwh.harvard.edu/pph2), (2) SIFT and (3) MutationTaster (www.mutationtaster.org) using the dbNSFP database. Filtration of common polymorphisms was accomplished using frequencies from the NHLBI Exome sequencing project (ESP) (http://evs.gs.washington.edu/EVS) 1,000 Genomes Project (VCF Version 4; www.1000genomes.org/wiki/Analysis/Variant%20Call%20Format/vcf-variant-call-format-version-41).

### Validation of Exome Sequencing Variants through Sanger Sequencing

Primers were designed using Oligo7 and Primer3 softwares for exome sequencing variants that were considered potentially relevant to the lymphedema phenotype. Polymerase chain reactions (PCRs) were performed using the Kapa HiFi HotStart ReadyMix DNA Polymerase (KapaBiosystems #KK2602) as per manufacturer's instructions. To visualize DNA fragments, 5 µL of the PCR product was loaded on a 1% agarose gel (or Lonza 1.2% agarose Flash Gel); ethidium bromide was used for staining. Purification of the DNA was done utilizing Qiagen Gel Extraction Kit (#28706) following manufacturer's instructions. Purified PCR products were sequenced at the University of Texas MD Anderson Cancer Center Genetic Core Facility using a 3730XL DNAnalyzer (AppliedBiosystems, Foster City, California, USA). Mutation *INPPL1* (O15357) p.T180A was validated using primers 5′ GAGGCCTTCTAAGACCCCAC 3′ and 5′ GGTGTAATACATGGGGCTGG 3′. Mutation *HGF* (P14210) p.G315V was validated using primers 5′ TGATCAGAAATCCACCTAGGGAT 3′ and 5′ ACATGTGGAGGTAAAATGCATTTAA 3′.

### Cell Culture

Primary human dermal lymphatic endothelial cells (HDLEC) were obtained from Science Cell (Carlsbad, CA) at passage 2 and cultured in complete endothelial cell media (ECM) supplemented with 5% fetal bovine serum (FBS), penicillin/streptomycin, and endothelial cell growth supplements (ECGS), from Science Cell. Human telomerase-immortalized dermal microvascular endothelial cells (TIME) were obtained from American Type Culture Collection (ATCC, Mannassas, VA) at passage 25 and cultured in complete endothelial growth medium, EGM-2MV, from Clonetics-Lonza (Walkersville, MD) supplemented with 5% FBS, EGF, VEGF, hFGF-B, R^3^-IGF-1, ascorbic acid, hydrocortisone and blasticin S HCl. Confirmation of lymphatic endothelial cell lineage was performed using flow cytometry analysis using anti-human podoplanin and CD31-fluorophore conjugated antibodies (eBioscience) and FACS data analyzed using FlowJo software (Treestar).

### RNAi transfections

Double-stranded ON-TARGET Plus human siRNA SHIP2-specific sequences (#1-UCAAGGAGCUUACGGAUCU; #2-GUCAGUACGUCCAGUGUGA; #3-CCAAGAAAGGGCUCUCAAA; #4-GCACACGUAUCGCAUUCUG and SmartPool of these 4-(Catalog# L-004152-00-005)) and ON-TARGET Plus Non-targeting (NT) Control (D-001810-01-05) were obtained from Dharmacon-Thermo Fisher (Lafayette, CO). Transfection was performed using Lipofectin reagent (Invitrogen), which is recommended for endothelial cells, according to manufacturer's instructions at a final concentration of 100 nM siRNA. To confirm transfection efficiency, Alexa Block-iT oligonucleotide (Invitrogen) was used. Mock transfection consisted of lipofectin reagent and Alexa Block iT oligonucleuotide only. Gene expression transcript and protein levels were determined using standard RT-qPCR and immunoblotting techniques.

### Site-directed mutagenesis

Human *INPPL1* TrueORF cDNA Myc-DDK clone was obtained from OriGene. SHIP2 T180A mutant in pCMV6-Entry-AC-MycDDK was made using QuickChange site-directed mutagenesis kit (Stratagene). Single nucleotides were changed at 71940153 of A→G (forward sequence) and T→C (reverse) resulting in T180A. Mutant plasmids were Sanger-sequenced at University of Texas MD Anderson Cancer Center Genetic Core Facility to confirm the introduction of point mutations before transfection into TIME cells. Single clones of transfected cells were selected for neomycin resistance using 200 µg/ml Geneticin (G418). Three clones from each transfection were analyzed and those that exhibited highest SHIP2 expression were selected for use in subsequent experiments and subsequently cultured using 400 µg/ml G418.

### MTS Cell Proliferation Assay

5×10^3^ cells/well were plated in 96-well plates and allowed to adhere overnight. For stimulation experiments, cells were starved 4 hours in 1% FBS, before growth factors were added. Cell proliferation was evaluated using tetrazolium salt colormetric assay (MTS) using CellTiter 96 AQeous One Solution (Promega) according to manufacturer's instructions at 24 hr, 48 hr and 72 hrs post plating.

### Cell Adhesion Assay

96-wells plates were coated with 1% BSA, 10 µg/cm^2^ collagen or 10 µg/ml bovine plasma fibronectin (Sigma) diluted in calcium/magnesium-free PBS overnight. Excess collagen and fibronectin was aspirated and all wells were blocked with 1% BSA for 2 hours, and then rinsed with PBS. 2×10^4^ cells/well were plated and allowed to adhere for 2 hours. Plates were washed 5 times with PBS to remove unattached cells, and adhered cells were then fixed using 4% paraformaldehyde, followed by permeabilization with 1% (w/v) Triton-X in PBS. Adhered cells were stained with 0.1% crystal violet in 2% ethanol and images acquired at 40× magnification.

### Wound-Healing Cell Migration Assay

Cells were plated in either 6-well plates or rectangular culture plates (Millipore) and when approximately 80–90% confluent and then perpendicular scratches created using either sterile P1000 pipette tips or cell combs (Millipore). Cells were rinsed with PBS, and incubated over 48 hrs, during which they were imaged using a Leica DM microscope (Leica Microsystems,) to assess the migration in closing the wound. Wound area was calculated using ImageJ software (NIH) as percentage of 0 hr coverage at wounding site.

### Tubulogenesis Assay

Matrigel matrix (Becton-Dickson) was liquefied overnight on ice at 4°C. 50 µl of the matrix was aliquoted in 96-well plates and incubated at 37°C for 30–60 min prior to plating 5×10^4^ cells per well. Cells were cultured for 24 hrs and tube formation monitored by inspection by phase contrast microscopy (Leica Microsystems). At the end of the 24 hr incubation, cells were labeled with 1 µM Calcein AM (Trevigen) and cell viability assessed using a fluorescent inverted microscope with a 485 nm excitation filter.

### AnnexinV Apoptosis Assay

Cells were plated in 6-well dishes, subjected to siRNA transfection for 48 hours then serum-starved overnight. Cells were treated with 1 µM staurosporine (Cell Signal) for 3 hours. Cells were harvested, washed in cold PBS, counted and apoptosis quantified using Alexa Fluor 488 AnnexinV/Propidium Iodide dead cell kit (Invitrogen) following manufacturer's instructions. Cells were analyzed by flow cytometry BD FACS Aria II using FITC and Texas Red filters and 50,000 events used for each sample. Data was analyzed using FlowJo Software (Treestar) and data presented as total AnnexinV positive % (sum of AnnexinV population representative of early apoptotic cells and double positive PI+AnnxV representing late apoptotic cells). Fluorescence mean intensity of FITC (AnnexinV) and phycoerythrin (PE) or PE-Texas Red (propidium iodide) is also presented.

### Proximity Ligation Assay and Immunofluorescence

Carefully following Duolink II instructions, cells were plated in chamber slides, allowed to adhere overnight, starved for 4 hours and stimulated with appropriate growth factors. Cells were then fixed using 4% paraformaldehyde (VWR) followed by permeabalization with 1% (w/v) Triton-X in PBS. Anti-DDK mouse monoclonal antibody (Origene) was combined with either rabbit polyclonals cMET or VEGFR3 (C28 and C20 respectively, SCBT). Following PLA protocol, cells were counterstained with DAPI and Alexa-Flour Phalloidin. Fluorescence spots were quantified using ImageJ software (NIH). For immunofluorescence, cells were serum-starved, stimulated, fixed and permeabilized as described above. Immunofluorescence cytochemistry was done using rabbit anti-SHIP2 antibody (Cell Signal) for determining expression of SHIP2 in LEC and anti-PIP3 IgG antibody (Echelon Biosciences) to determine cellular levels of PtdIns(3,4,5)P3. Cells were counterstained with either Alexa Flour 488 or 594 phalloidin (Invitrogen) to show actin localization and either DRAQ5 (Biostatus) or DAPI (Invitrogen) to show cell nucleus. Cells were visualized using either confocal laser microscope (Leica), epifluorescence inverted microscope (Leica) or EVOS FL microscope (Invitrogen).

### Malachite Green Phosphatase Assay

Cells were serum-starved overnight then lysed using a nondenaturing buffer (10 mM sodium phosphate pH 7.5, 150 mM NaCl, 1 mM PMSF and 1X Halt Protease and Phosphatase inhibitors cocktail). FLAG antibody was preabsorbed overnight with Protein A/G sepharose beads (SantaCruz Biotechnology) and immunocomplexed with precleared 500 µg lysates. Immunocomplexes were washed and FLAG-tagged protein eluted from agarose beads using 2 M glycine buffer, pH 2.8, and protein concentration quantified using BCA assay (Pierce). Eluted SHIP2 (0.05 µg) was combined with 120 µM recombinant PI(3,4,5)P3 substrate (P-3908, Echelon Biosciences) at final concentrations of 0.025 µg and 60 µM, respectively and catalysis allowed to occur at 37°C for 30 mins. Reactions were stopped using BIOMOL GREEN (AK-111, Enzo Biosciences), incubated at room temperature for 15 mins while shaking, and absorbance read at 620 nm. Phosphate standard curve was performed to estimate the concentration of released phosphate in the experimental samples. 0.05 µg recombinant SHIP2 (Echelon Biosciences) was used as positive control and 120 µM recombinant PIP3 was used as negative control (substrate only, no enzyme).

### Immunoblotting, Immunoprecipitation and PIP3 delivery

Cells were grown to desired confluency and lysed in buffer (20 mM Tris pH 9, 137 mM NaCl, 5 mM EDTA and 0.5% (w/v) SDS containing 1 mM phenylmethylsulfonyl fluoride (PMSF) and 1X Halt Protease and Phosphatase inhibitors cocktail (Thermo Fisher). Protein concentration was measured using BCA assay kit (Pierce). 50 µg total protein was loaded in 4–20% TGX SDS-PAGE gels (BioRad), and Western blots probed with appropriate primary antibodies (goat polyclonal SHIP2 (I-20, SCBT); rabbit antibodies against SHIP2, DYKDDDDK, pAKT-S473, pAKT-T308, pP44/P42, AKT, P44/P42, COX IV, β-actin and GAPDH from Cell Signal). For signaling studies, after 48 hr siRNA transfections, cells were serum-starved for 4 hrs and stimulated with 30 ng/ml rmHGF or 100 ng/ml rhVEGFC (R&D Systems) in 1% FBS prior to lysis and immunoblotting. For inhibitor studies, cells were pretreated with 10 µm U0126 for 1 hour or 50 µm LY294002 for 2 hours prior to growth factor stimulations. For AS1949490 SHIP2 inhibitor studies, cells were serum-starved overnight then treated with increasing doses of AS1949490 in starvation media for 1 hour prior to growth factor stimulations. For immunoprecipitations, cells were serum starved overnight and stimulated with indicated growth factors and lysed with IP lysis buffer (25 mM Tris-HCl pH 7.4, 150 mM NaCl, 1 mM EDTA, 1% NP-40 and 5% glycerol contianing1mM PMSF) followed by centrifugation to remove debris. 1 µg IP antibody was pre-adsorbed with 20 µl Protein A/G PLUS agarose beads (Santa Cruz) overnight and washed with PBS and blocked with 10% BSA. 500 µg total lysates were complexed with pre-adsorbed antibody-beads for 4 hours at 4°C and immunocomplexes washed with cold PBS before samples were denatured and subjected to electrophoresis and immunoblotting. For exogenous PIP3 delivery, cells were treated for 1 hour with increasing concentrations of recombinant PtdIns(3,4,5)P3 using Shuttle PIP kit (Echelon Biosciences) following manufacturer's instructions before proceeding with standard immunoblotting techniques. All blots were followed by IRDye-labeled secondary antibodies (LICOR), and blots were viewed using Odyssey Classic fluorescence viewer (LICOR).

### Boyden Chamber Chemotaxis Assay

Boyden chamber assay was performed using ThinCerts (3 µm pores, Grenier BioOne) precoated with 1 ug/ml fibronectin before use. Cells were starved overnight before seeding into upper chambers (2×10^5^/well) in 1% FBS and stimulant media added to the bottom wells. Chemotaxis was assessed after staining with DRAQ5 by measuring fluorescence of cells migrated to the lower chamber using EVOS FL microscope using filters set at 618/40 nm excitation and 692/40 nm emission. Number of migrated cells was quantified using ImageJ (NIH).

### Statistical analysis

The statistical analysis was performed using SigmaPlot 11.0. Student t-test and one way ANOVA were used to evaluate the data. Data are expressed as means ± SEM. All *in vitro* experiments independently repeated at least 3 times. *p* values of <0.05 were considered statistically significant.

## Results

### NIRF lymphatic imaging of subjects in a nucleus family with variable lymphedema phenotypes

As illustrated in **Fig 1A–D** and **Videos S1** and **S3**, healthy lymphatics are typically linear and well-defined, without lymphatic abnormalities such as dermal backflow, hyperplastic lymphatic capillaries radiating from injection sites, dilated vessels, or tortuous lymphatics typically observed in diseased subjects [25], [26]. We conducted lower extremity NIRFLI and collected DNA from a nucleus family of four (**Fig 1E–J**) harboring two probands, sisters aged 32 and 37 years old at the time of imaging, who were diagnosed with congenital lymphedema of the lower extremities and with *praecox* lymphedema of the left lower extremity, respectively. There were no other siblings in the nucleus family, but with next generation sequencing and cosegregation bioinformatics analyses, as little as four members are needed to discover new disease causative gene variants [27], provided phenotyping with NIRFLI could identify affected family members. Their 58 year old father (Subject #3) had no reports of swelling or any other clinical signs of lymphedema. NIRFLI revealed that his lymphatics appeared normal, with the exception of a few lymphatic capillaries radiating from the injection sites on the left foot (**Fig 1E**) and medial calf. At the time of study, the 59 year old mother (Subject #4) had not been diagnosed but presented with edema of both ankles, self-reported to initially occur following a minor sprain on her left ankle at 54 years old thus swelling on her right ankle was spontaneous. Upon NIRF imaging and medical examination, her clinical diagnosis was classified as acquired (secondary) lymphedema on the right leg and *en tarda* lymphedema on the left leg. NIRFLI revealed some additional abnormalities including fluorescent lymphatic capillaries radiating from the injection sites of the left foot (**Fig 1F**), dermal backflow on the medial left ankle (**Fig 1G**) and tortuosity on the right foot and right medial knee. She also possessed few varicose veins.

The 37 year old daughter (Subject #1) had been diagnosed with Grade I unilateral lymphedema *praecox* at 14 years of age and possessed generally well-defined lymphatics in the right leg, with the exception of several fluorescent lymphatic capillaries radiating from the injection sites on the foot with some retrograde flow towards the toes (**Video S2**). Her right “unaffected” shin also exhibited a lymphatic vessel crossing over just below the knee (**Video S4**). However, her left edematous foot exhibited dermal backflow (**Fig 1H**) with fluorescent lymphatic capillaries radiating from the injection sites on the medial ankle and calf. The 32 year old daughter (Subject #2) had been diagnosed with Grade II bilateral lower extremity, congenital lymphedema at 1 year of age. NIRFLI revealed extensive dermal backflow in both legs as shown in the right foot (**Fig 1I**), the right ankle and the left lateral calf (**Fig 1J**). In summary, the sister diagnosed with congenital lymphedema (Subject #2) had few, if any, functional lymphatics resulting in extravascular deposition of ICG. In both subjects, other than edema and minimal toe fibrosis and skin cellulitis, there were no additional remarkable clinical signs such as varicose veins, distichiasis, limb hypertrophy, or vascular malformations.

### Identification of a rare *INPPL1* mutation in a large family with varied lymphedema phenotypes

Whole exome sequencing (WES) results showed both probands from the nucleus family possessed a rare, heterozygous, damaging SNP within the alpha-chain of *HGF* (81359017 Hg19 resulting in C>A; p.G315V), but not in any other gene previously associated with lymphatic dysfunction. In their gene candidate studies, Finegold and coworkers [28] found *HGF* and *cMET* mutations in single subjects with primary lymphedema, lymphangiectasia, and breast cancer-related lymphedema (BCRL) subjects that were not polymorphic in control populations. They concluded that *HGF* and *cMET* mutations were likely causal for primary lymphedema and/or susceptibility genes for acquired lymphedema, an affliction that impacts an estimated 15% of all cancer survivors [29]. HGF and cMET are both important for lymphatic vessel formation [30]. Additionally, because Saito *et al.* [31] showed that HGF gene therapy reduced lymphedema volume in the rat tail lymphedema model created by lymphatic vessel disruption, we expected the mother to harbor the *HGF* gene mutation, making her susceptible to her diagnosed acquired or *en tarda* form of lymphedema. However, she lacked the *HGF* gene variant, and possessed an abnormal NIRFLI phenotype. Surprisingly, the asymptomatic father who did not possess an abnormal NIRFLI phenotype had passed the *HGF* gene variant to his daughters.

As no other lymphedema-related genes were identified in this family, we conducted WES bioinformatics analyses to identify rare SNPs which could be likely causative or could increase susceptibility to lymphedema. Given that HGF could not explain lymphatic disease in the mother (Subject #4), and the varied penetrance of lymphedema in this family, we conducted pathway analysis with the goal of identifying potential HGF-modifier genes that could have been inherited from the mother that contribute to the probands' disease. We identified a rare, heterozygous missense mutation in *INPPL1*, which encodes SHIP2, in the probands (Subjects #1 and #2) and in their mother (Subject #4), but not in their father (Subject #3). This *SHIP2* mutation results in the conversion of threonine 180, located carboxyl to the SH2 domain, to alanine (p.T180A) (**Fig S1A**). The T180A *INPPL1* mutation was validated by Sanger sequencing (**Fig S1B**). We did not confirm that mRNAs encoding T180A SHIP2 protein was produced in affected individuals nor RNAseq since the tissue biopsy required for RNAseq is well-known to be associated with an increased risk for infection in lymphedema subjects. While an *INPPL1* SNP at 71940154 (C>G; Hg19) resulting in T180S has been previously reported (rs376749049), our identified SNP at 71940153 (A>G; Hg19) resulting in T180A, has not been reported in SNP databases. The genomic region that harbors the *INPPL1* gene is unique, in particular the area surrounding the T180A mutation. To verify this mutation was not in an intronic region, we performed a blast analysis ± 100 bp on each flanking region, and there was a single hit on *INPPL1*. NM_001567 is the single transcript for *INPPL1* and there are no alternative splice versions, in addition RNAseq experiments have been used to identify the correct exons described in the gene model. The mutation that we describe in Hg19 chr11:71940153 corresponds to the codon 180 of exon 5 of SHIP2. This particular region is evolutionary conserved, free of repeat elements and with no common SNPs reported, consequently we consider *INPPL1* an evolutionary conserved gene with low tolerance for mutations.

**Figure 2:**
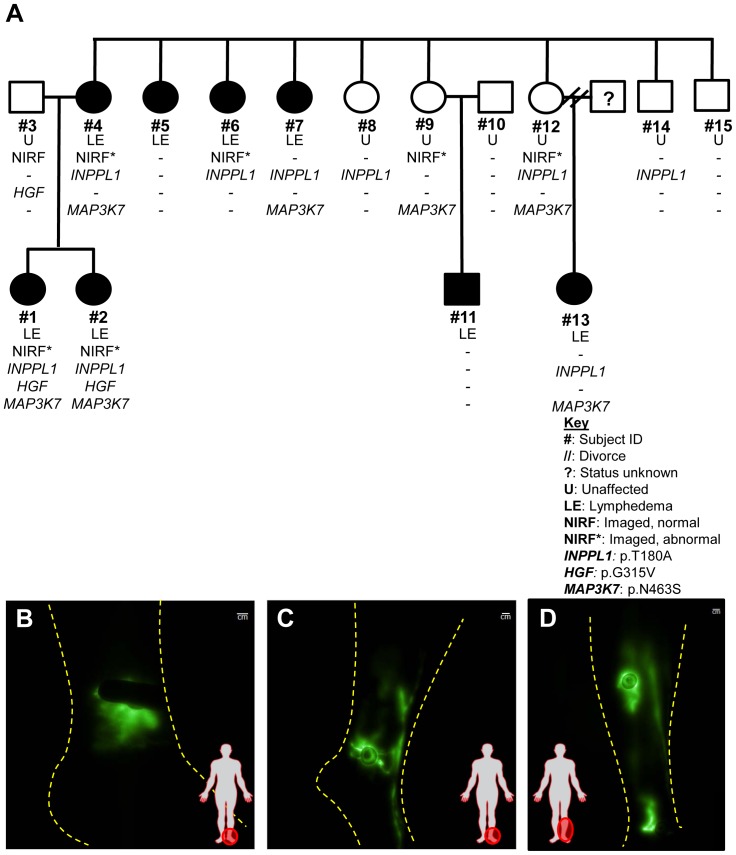
Identification of T180A SHIP2 mutation in familial lymphedema. (**A**) Pedigree of the nucleus and extended family showing affected (filled) and unaffected (open) subjects with lymphedema, phenotyped by NIRF imaging and WES analysis revealing subjects who harbor T180A *SHIP2*, G315V *HGF* and N463S *MAP3K7* mutations. NIRF imaging reveals (**B**) dermal backflow in medial left ankle of Subject #6 (bilateral lymphedema *praecox*) and also has abnormal lymphatic capillaries on thigh (not shown). (**C**) Abnormal lymphatic capillaries radiating from the injection site on the medial left ankle of Subject #9 which were also observed capillaries on the thigh on this unaffected subject. (**D**) Tortuous lymphatics draining the medial left ankle and lymphatic capillaries radiating from the left medial calf in Subject #12 (unaffected). All three subjects also appeared to have dilated lymphatics.

Given the knowledge that there were other extended family members with lymphedema, we further conducted NIRFLI and/or WES studies on 11 additional maternal family members. Out of the 11 extended family subjects, excluding the nucleus family, 5 subjects (Subjects #5, 6, 7, 11, and 13) were previously diagnosed with lymphedema (**Fig 2A**). Seven subjects (including nucleus family) were phenotyped with NIRFLI and genotyped with WES, of which 5 subjects harbored the T180A SHIP2 mutation, which was validated by Sanger sequencing. Of those 5, 4 subjects (Subjects #1,2,4 and 6) were diagnosed lymphedema and 1 subject (Subject #12) was unaffected but her NIRFLI phenotype revealed vessel tortuosity, fluorescent lymphatic capillaries radiating from injection sites, and dilated vessels (**Fig 2D**). Her 30 yr old daughter (Subject #13) was diagnosed with bilateral lower extremity *praecox* lymphedema at 13 yrs and also harbored T180A SHIP2. No clinical information was available for Subject #13′s father. Subject #6 had swollen ankles and arms with venous insufficiency, and NIRF imaging revealed dermal backflow in her medial left ankle with diffuse dye uptake (**Fig 2B**), fluorescent lymphatic capillaries radiating from injection sites on thighs and limited lymphatic propulsion. The lymphatics of Subject #9 (unaffected) were mostly well defined but we observed slight tortuosity with fluorescent lymphatic capillaries radiating from injection site on medial ankle (**Fig 2C**). All 15 subjects were genotyped with WES, and no other lymphedema-related genes were identified, however a mutation of a gene encoding a protein within the MAPK/ERK pathway (*MAP3K7*) was identified in Subjects# 1, 2, 4, 7, 9, 12 and 13. Out of these 7 subjects with *MAP3K7* mutation (Hg19 chr6:91229032; T> C) 5 of them were diagnosed with lymphedema (except Subjects #9 and 12) and also harbored the T180A SHIP2 mutation. The rare *MAP3K7* SNP, which results in p.N463S, has been reported in SNP databases (rs375179983), but has not yet been associated with any human disease. The frequency of the *MAP3K7* mutation within 6500 exomes is very low (0.01%) and the resultant protein change is predicted to the functionally damaging by PolyPhen-2 software. Of the total 15 subjects, 8 subjects were not imaged by NIRFLI and thus are considered incomplete phenotypes even though they were sequenced. Out of these 8 subjects, 4 were diagnosed with lymphedema, of which 3 have T180A SHIP2. In Subjects #5 and #11, SHIP2 has no contributory role to their lymphedema but both subjects had later onset of symptoms (27 yrs and 47 yrs respectively) and additional lymphatic insults which either exacerbated (breast cancer surgery in Subject #5) or triggered edema (self-reported>14 hour long flight in Subject #11). Two subjects (Subjects #8 and #14) were unaffected by symptoms, yet harbor the T180A SHIP2 mutation, however, it remains unknown whether these have any NIRFLI lymphatic phenotype or will encounter symptoms upon an injury, as did Subject #4. All subjects with lymphedema had lower extremity disease, except Subject #6 who had both upper and lower extremity lymphedema. There were no other lymphatic abnormalities such as chylous ascites or chylothorax reported in this family. Results are summarized in **Table 1**.

**Table 1:** Summary of lymphedema diagnosis and T180A SHIP2 status of nucleus and extended family shown in Fig 1 and 2.

	Relation	Subject ID	*INPPL1* p.T180A	Lymphedema Diagnosis	Onset of Symptoms	Age at Study	NIRF Phenotype	Comments
Nucleus Family	Proband	#1	Yes	*Praecox*	14	37	Abnormal	Diagnosed Grade I left unilateral lower extremity LE on dorsum of left foot. Minimal fibrosis at base of toes
	Proband	#2	Yes	Congenital	1	32	Abnormal	Diagnosed Grade II bilateral lower extremity LE from knees down. Skin cellulitis.
	Father	#3	No	Unaffected	N/A	58	Normal	N/A
	Mother	#4	Yes	*en tarda (*left) Acquired (right)	54	59	Abnormal	Diagnosed Grade I right leg; Grade II left leg. Minor left ankle sprain preceded symptoms on both legs. Few varicose veins.
Extended Family	Maternal Aunt	#5	No	*Praecox*	27	58	N/A	Bilateral LE in both legs, ankles and feet. Mild edema in both arms following breast cancer bilateral lymph node dissection surgery. Venous insufficiency
	Maternal Aunt	#6	Yes	*Praecox*	8 (legs) 15(hands)	63	Abnormal	Bilateral LE in both legs, both hands and arms. Visible enlarged veins and telangiectasia. Venous insufficiency
	Maternal Aunt	#7	Yes	*Praecox*	20	61	N/A	Bilateral LE in both legs, feet and toes
	Maternal Aunt	#8	Yes	Unaffected	N/A	50	N/A	N/A
	Maternal Aunt	#9	No	Unaffected	N/A	69	Some abnormality	N/A
	Uncle (by marriage)	#10	No	Unaffected	N/A	77	N/A	N/A
	Maternal Male Cousin	#11	No	*en tarda*	47	50	N/A	Unilateral left foot LE; long>14 hr flight preceded symptoms.
	Maternal Aunt	#12	Yes	Unaffected	N/A	54	Abnormal	N/A
	Maternal Female Cousin	#13	Yes	*Praecox*	13	30	N/A	Mild edema in both legs below the knee.
	Maternal Uncle	#14	Yes	Unaffected	N/A	48	N/A	N/A
	Maternal Uncle	#15	No	Unaffected	N/A	55	N/A	N/A

In summary of these clinical studies, the results of WES analysis combined with NIRFLI phenotyping suggested that the T180A SHIP2 mutation contributes *to but does not necessarily cause* lymphatic dysfunction. Furthermore, these analyses shows that in this large extended family, lymphatic abnormalities are more severe and of earlier onset in individuals with additional mutations in genes that have been associated with lymphatic disease previously, such as *HGF*, or within MAPK/ERK signaling pathway [28], [32]. Based on these findings as well as previous reports that SHIP2 physically associates with cMET [33], we investigated a potential functional role for SHIP2 and mutant T180A-SHIP2 in LEC.

#### Dysregulated HGF- and VEGFC-induced activation of AKT and MAPK in SHIP2-deficient LEC

**Figure 3:**
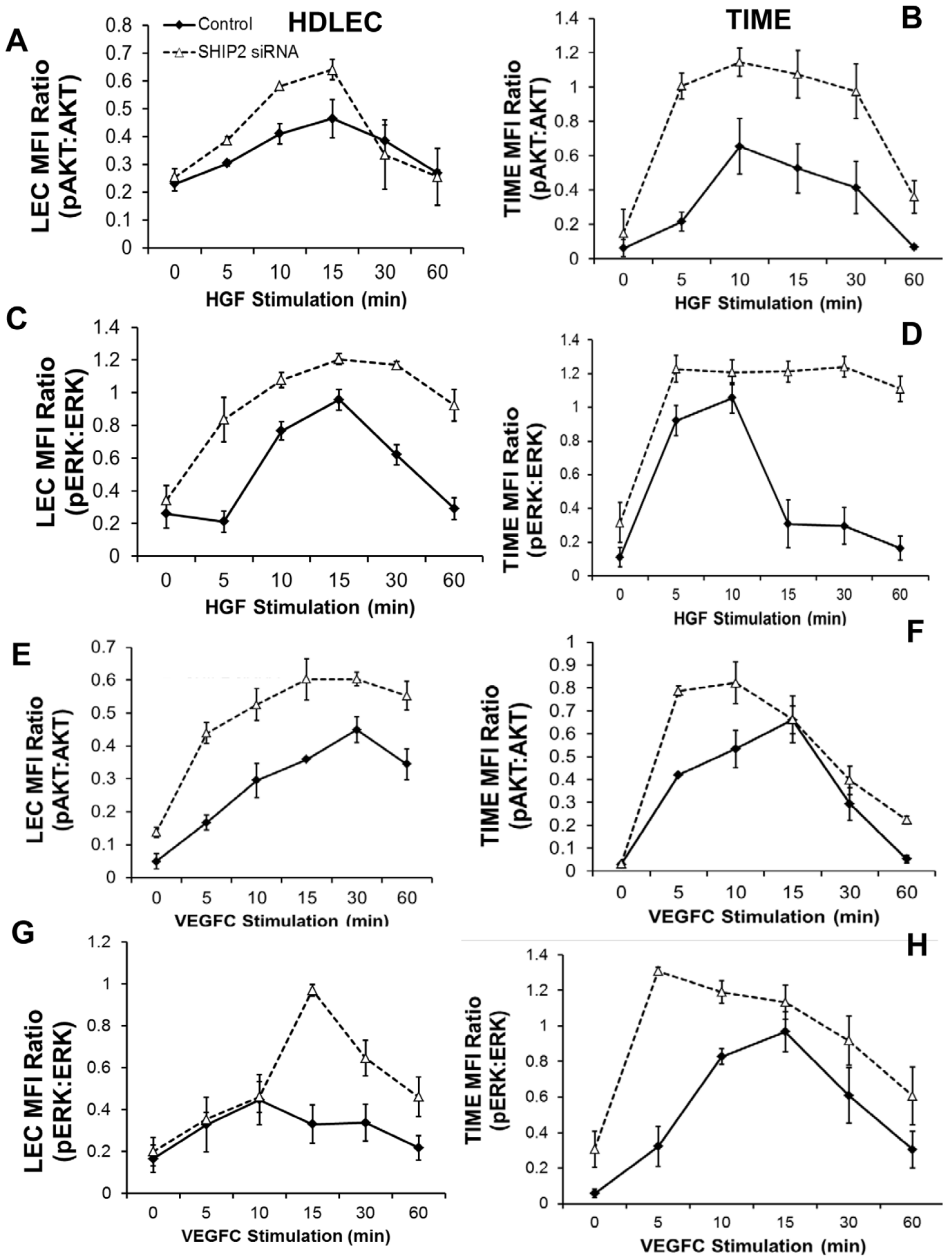
Dysregulated HGF- and VEGFC-induced activation of AKT and ERK1/2 in SHIP2-deficient LEC. HDLEC (**A,C,E,G**) and TIME (**B,D,F,H**) were subjected to 48 hr SHIP2 siRNA and stimulated with HGF (**A–D**) or VEGFC (**E–H**) for the indicated times. Activation of AKT and ERK was determined by fluorescent double staining Western blotting of cell lysates with both phosphospecific antibodies and antibodies to total proteins. (See **Fig S4** for representative blot images) Quantification of AKT (**A,B, E,F**) and ERK (**C,D,G,H**) activation in LEC and TIME by average mean fluorescence intensity (MFI) and represented as a ratio of pAKT-S473 to total AKT and pERK1/2 to total ERK1/2, respectively. Data presented as mean±SEM of MFI from 4 independent experiments.

The role of SHIP2 has been previously evaluated in other cell types but not in LEC [22], [33], [34]. We confirmed the lymphatic lineage of primary human dermal lymphatic endothelial cells (HDLEC) and TIME (telomerase-immortalized microvascular endothelial) cells by flow cytometric analysis of lymphatic-specific podoplanin and pan-endothelial CD31 antigen expression (**Fig S2A**). We found that SHIP2 was expressed in HDLEC (**Fig S2B**) and in TIME cells (not shown) *via* immunofluorescence. Given that a major reported function of SHIP2 is to dampen PI3K/AKT signaling, and that aberrant PI3K/AKT signaling results in lymphatic dysfunction in both mouse [18]–[20] and humans [15], [16], we examined the effect of SHIP2 knockdown upon HGF- and VEGFC-induced AKT activation in LEC. Transfection of HDLEC and TIME with SHIP2 siRNA resulted in effective knockdown of SHIP2 as revealed by immunofluorescence (**Fig S2B**), Western blotting, and RT-qPCR (**Fig S2C,D,E**). Activation of AKT in HDLEC and TIME was examined by Western blotting using phospho-specific anti-AKT antibodies (**Fig 3A,B,E,F**). Stimulation of control HDLEC resulted in activation of AKT with the peak response evident between 10 and 15 minutes and 10 and 30 minutes after stimulation with HGF and VEGFC, respectively (**Fig 3A, 3E** **and Fig S3A, S3C**). After SHIP2 knockdown HDLEC, the magnitude of AKT activation was modestly increased in response to HGF and substantially increased in response to VEGFC, although the kinetics of responses were not altered. Similar results were obtained in TIME cells after SHIP2 knockdown (**Fig 3B, 3F** **and Fig S3B, S3D**). These data are consistent with a function for SHIP2 as a negative regulator of the PI3K/AKT signaling pathway in LEC.

Recent studies have also identified mutations within the Ras/MAPK/ERK signaling network as responsible for lymphatic abnormalities [13], [17], [32], [35]–[37]. Given that we identified a mutation in *MAP3K7*, a Ras/MAPK/ERK-related gene, we also examined activation of MAPK in SHIP2 knockdown HDLEC using phospho-specific anti-ERK antibodies. In control HDLEC, HGF and VEGFC both induced activation of ERK with peak responses occurring at 15 and 10–15 minutes post-stimulation respectively. In SHIP2 knockdown HDLEC, substantial increases in the magnitude of ERK activation were evident in response to HGF and VEGFC stimulation. In both cases, activation of ERK occurred earlier and persisted for longer after growth factor stimulation (**Fig 3C, 3G** **and Fig S3A, S3C**). These results were replicated in TIME cells in which we observed potent and protracted ERK activation upon SHIP2 knockdown in response to both HGF and VEGFC stimulation (**Fig 3D, 3H** **and Fig S3B, S3D**). This data implicates SHIP2 as a negative regulator of MAPK signaling, as well as negative regulator of PI3K/AKT activation in LEC.

### SHIP2 is required for HGF- and VEGFC-induced lymphangiogenesis *in vitro*

**Figure 4:**
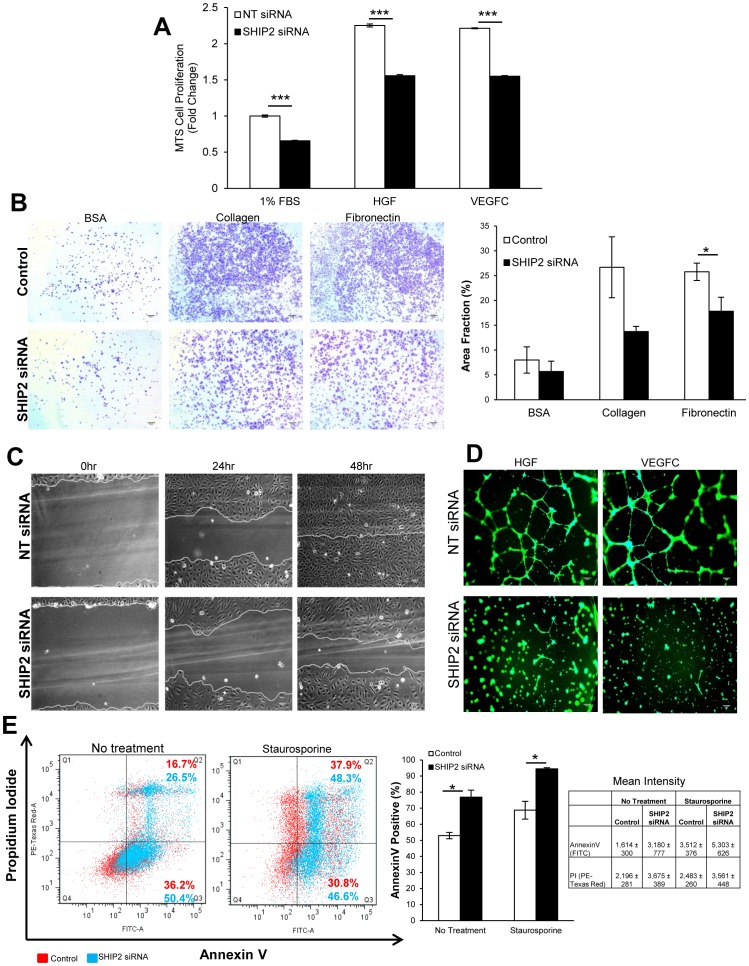
SHIP2 is required for *in vitro* lymphangiogenesis in primary HDLEC. (**A**) MTS cell proliferation assay in response to growth factor stimulation for 48 hrs, normalized to 1% FBS of non-targeting (NT) siRNA (N = 3). (**B**) Cell adhesion assay of siRNA-transfected HDLEC onto BSA, collagen and fibronectin and quantification presented as area fraction of whole images (triplicates per experiment; 5 independent experiments). (**C**) Wound scratch cell migration assay of siRNA-transfected HDLEC immediately after injury, at 24 hr and 48 hrs afterward. (**D**) 3D tube formation networks in response to growth factors imaged 24 hrs post plating. (**E**) Annexin V apoptosis assay of siRNA-transfected HDLEC either untreated or following staurosporine treatment, quantification of total AnnexinV-positive cell populations and mean intensity of AnnexinV and propidium iodide of 3 independent experiments. Data presented as means±SEM. *p<0.05, ***p<0.001. Scale bar  = 50 µm. Control  =  untransfected cells; NT = non-targeting siRNA.

We next sought to investigate the role of SHIP2 in lymphangiogenesis using *in vitro* assays of cell proliferation, adhesion, migration, and tubulogenesis that model different steps in the lymphangiogenic response. Given the increases in AKT activation (**Fig 3** **and Fig S3**), we expected SHIP2 knockdown to result in increased LEC proliferation. However, we found reduced LEC proliferation both under basal conditions (1% FBS) and following HGF or VEGFC stimulation (**Fig 4A**). SHIP2-deficient LEC also exhibited a reduced capacity to adhere to the extracellular matrix proteins, collagen, and fibronectin (**Fig 4B**) and showed reduced directed migration in scratch wound assays (**Fig 4C**). The ability of LEC to organize into tube-like structures was assessed using a 3D-Matrigel assay. SHIP2 knockdown resulted in significantly less tube formation after HGF and VEGFC stimulation (**Fig 4D**). However, contrary to the known pro-survival role of PI3K/AKT, we found increased apoptosis in SHIP2 knockdown LEC induced by overnight serum starvation or staurosporine treatment (**Fig 4E**). The same results were obtained in TIME cells in all functional assays (**Fig S4**).

Taken together, these data suggest that while SHIP2 is necessary for *in vitro* lymphangiogenesis in HDLEC and TIME, the cellular phenotypes of reduced proliferation, adhesion, and migration may be secondary to increased apoptosis. To verify that the observed pheonotype was not as a result of reagent toxicity, tube networks were stained with Calcein AM viability fluorescent dye (**Fig 4D**) and four SHIP2 RNAi with different targeting sequences were tested, including mock and non-targeting (NT) control transfections, and all four RNAi oligonucleotides showed comparable levels of SHIP2 knockdown (**Fig S2C–S2D**). Additionally, pharmacological inhibition of SHIP2 using AS1949490, a selective inhibitor of SHIP2 phosphatase function without affecting protein levels, results in dose dependent activation of AKT and MAPK and suppresses *in vitro* lymphangiogenic responses further confirming that the observed siRNA phenotype seen herein are not due to off target effects, but rather due to alteration of SHIP2 (Agollah *et al.* 2014). Why loss of SHIP2 would lead to increased AKT activation but increased apoptosis of LEC is unclear but may be explained by still greater increases in pro-apoptotic ERK activation (see Discussion).

### Effect of T180A SHIP2 mutation in functional responses in LEC

**Figure 5:**
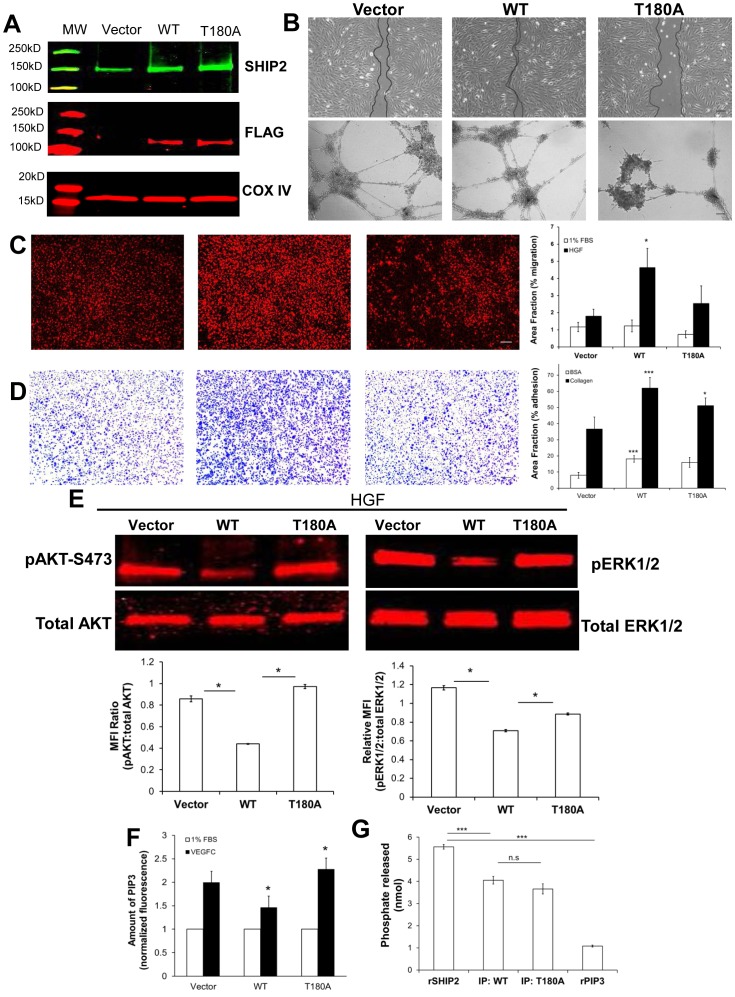
Influence of T180A-SHIP2 on LEC functional responses. (**A**)Western blot analysis depicting expression of SHIP2 (green) and DYKDDDDK (FLAG) tag epitope (red) in SHIP2-FLAG TIME transfectants. COX IV (red) used to determine equal protein loading. (**B**) T180A-SHIP2 exhibits reduced migration assessed by wound healing (top panels) and tube formation (bottom panels). (**C**) WT-SHIP2 exhibits increased chemotaxis compared to Vector in response to HGF while T180A-SHIP2 has slightly reduced, but not significant, chemotaxis towards HGF, compared to WT. Data are presented as % of area covered by migrated cells on underside of transwell filters and cells stained with DRAQ5. (**D**) WT-SHIP2 exhibits increased adhesion (assessed by crystal violet staining) over Vector on BSA and collagen while T180A-SHIP2 shows decreased adhesion to collagen compared to WT. (**E**) T180A-SHIP2 results in increased activation of AKT and ERK upon HGF stimulation in comparison to WT-SHIP2. Representative blots shown. Phosphorylation was determined by Western blotting of cell lysates with phosphospecific antibodies. Blots were reprobed with total AKT and ERK antibodies to demonstrate equal loading. Phospho- and total-antibody reprobes were detected with IRDye 680 nm secondary antibodies. Quantification of AKT and ERK activation performed by average mean fluorescence intensity (MFI) of 3 independent experiments and represented as a ratio of pAKT-S473 to total AKT and pERK1/2 to total ERK1/2, respectively. (**F**) WT-SHIP2 results in less VEGFC-induced PIP3 levels compared to Vector, while T180A has increased PIP3 levels compared to WT and data expressed as fluorescence intensity of PIP3 normalized to 1% FBS in 100 cells/experiment (N = 3). PIP3 levels were assessed by fluorescent immunocytochemistry of 10 min VEGFC-stimulated cells using anti-PIP3 antibody. (**G**) Malachite green *in vitro* phosphatase assay indicates no significant difference in amount of phosphate released by T180A SHIP2 over WT-SHIP2 suggesting similar enzymatic activity against recombinant PIP3 substrate. Recombinant SHIP2 (rSHIP2) and PIP3 (rPIP3 only, no enzyme) used as positive and negative controls respectively. Comparisons are between Vector *vs*. WT; WT *vs*. T180A and rSHIP2 *vs.* WT; and rSHIP2 *vs.* rPIP3 *p<0.05, **p<0.01, *** *p*<0.001. Scale bar  = 100 µm (**B**) and 50 µm (**C** and **D**) MW =  molecular weight marker.

We next examined whether the T180A mutation affected the function of SHIP2 in a cellular context. FLAG-tagged empty vector, wild type (WT) SHIP2 and T180A SHIP2 mutant were stably expressed in TIME cells. As revealed by Western blotting using a FLAG antibody, WT and mutant SHIP2s were comparably expressed (**Fig 5A**). As shown above, SHIP2 knockdown resulted in reduced LEC proliferation, adhesion, directed migration, tube formation, and survival (**Fig 4** **and Fig S4**). However, over-expressed WT SHIP2 did not largely influence LEC proliferation, survival (data not shown for brevity) or tube formation (**Fig 5B**), although overexpression of WT SHIP2 did result in increased LEC migration, adhesion to collagen and increased chemotaxis in response to HGF (**Fig 5B–5D**). On the other hand, overexpressed T180A SHIP2 resulted in decreased migration, tube formation, chemotaxis to HGF, and adhesion to collagen in comparison to overexpressed WT SHIP2 (**Fig 5B–5D**), suggesting a loss of function cellular phenotype similar to siRNA studies (**Fig 4**).

Consistent with its role as a negative regulator of PI3K/AKT signaling, over-expression of WT SHIP2 in TIME cells resulted in reduced HGF- induced AKT activation compared to TIME cells that were transfected with vector alone (**Fig 5E**). However, this inhibitory effect of over-expressed WT SHIP2 upon HGF-induced AKT activation was abrogated by the T180A SHIP2 mutation (**Fig 5E**). We also assessed the effect of over-expressed WT and mutant SHIP2 upon ERK activation in TIME cells. In agreement with results obtained in knockdown experiments (**Fig 3** **and Fig S3**), over-expression of WT SHIP2 resulted in reduced HGF -induced activation of ERK (**Fig 5E**). However, the T180A mutation abolished an ability of over-expressed wild type SHIP2 to inhibit ERK activation in response to HGF. Similar results were seen in VEGFC-induced activation of AKT and ERK, whereby WT SHIP2 reduced activation while T180A SHIP2 failed to dampen VEGFC-induced activation of AKT and ERK (data not shown for brevity). Given findings of increased AKT activation by T180A (**Fig 5E**), we examined whether T180A SHIP2 mutation might affect levels of PI(3,4,5)P3, the PI3K product. 10 min VEGFC treatment decreased PIP3 levels in WT SHIP2, compared to Vector controls, analyzed with fluorescence immunocytochemistry of anti-PIP3 antibody, while T180A resulted in increased PIP3 levels following stimulation, compared to WT SHIP2 (**Fig 5F**). The T180A mutation resides between the SH2 and catalytic (5′-phosphatase) domains. Because the entire crystal structure of the SHIP2 protein has not been solved, the potential impact of the T180A mutation on intramolecular interactions that could impact upon catalytic and non-catalytic (scaffolding) activity could not be modeled. However, we examined the potential impact of T180A mutation on catalytic phosphatase activity directed toward recombinant PIP3 in i*n vitro* phosphatase assays. While VEGFC-induced PIP3 cellular levels were increased in T180A (**Fig 5F**), phosphatase activity, measured by *in vitro* green malachite assay, of T180A SHIP2 against recombinant PIP3 was reduced but not statistically different compared to WT SHIP2 in this assay (T180A's 3.7 nmol *vs*. WT's 4.1 nmol released phosphate; *p* value = 0.202).

### SHIP2 physically interacts with cMET and VEGFR3 in LEC

**Figure 6:**
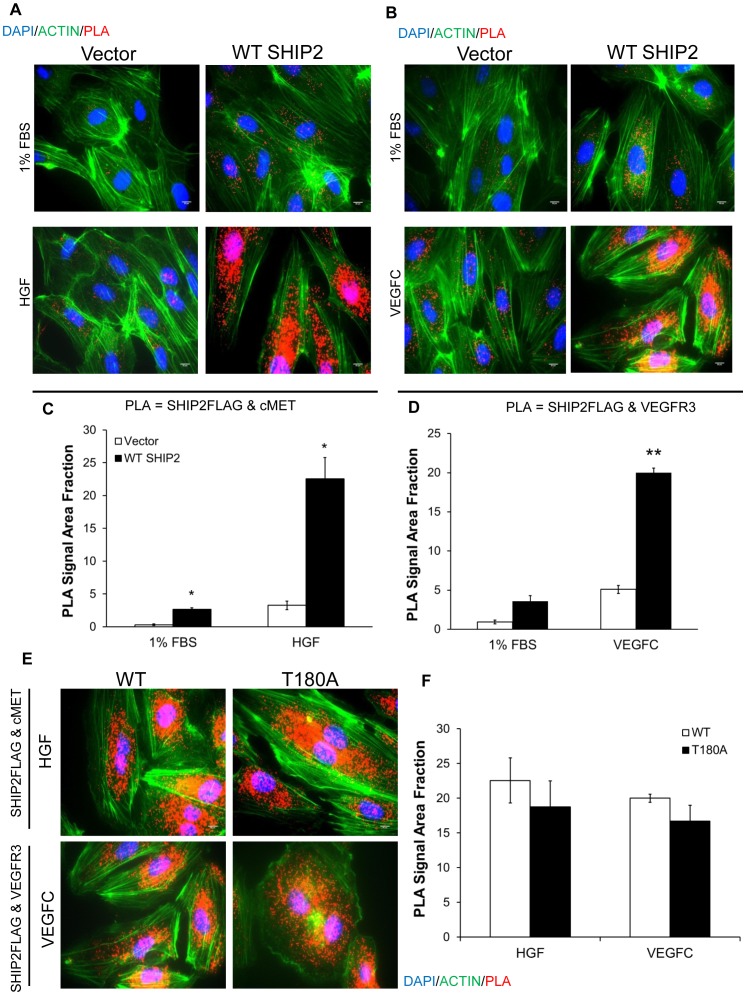
SHIP2 interacts with cMET and with VEGFR3. Proximity ligation assay (PLA) depicting interaction of (**A**) SHIP2-FLAG and cMET and (**B**) SHIP2-FLAG and VEGFR3 in Vector- and FLAG-tagged WT SHIP2-transfected TIME cells following (**A**) HGF stimulation and (**B**) VEGFC stimulation and respective quantification of PLA fluorescence signal (**C** and **D**) in 100 cells/experiment (N = 3) quantified by ImageJ. (**E, F**) No statistical difference is seen between WT SHIP2 *vs*. T180A SHIP2 in their interaction with cMET and with VEGFR3. Quantification of 100 cells/experiment (N = 3). Blue  =  DAPI, green  =  actin and red  =  PLA fluorescence with each red dot depicting *in situ* interaction site. *p<0.05, **p<0.01. Scale bar  =  50 µm.

SHIP2 interacts physically with cMET in epithelial cells [33]. To confirm this interaction in LEC and investigate whether SHIP2 also interacts with VEGFR3, we used proximity ligation assays (PLA) [38]. PLA allows for *in situ* detection and quantification of protein-protein interactions in intact cells, whereby a fluorescent signal is generated when two PLA probes are present in close proximity. Through transfection of FLAG-tagged WT SHIP2 into TIME cells we confirmed that SHIP2 interacts with endogenous cMET upon HGF stimulation in PLA assays (**Figure 6A**; PLA signal from interaction of FLAG and cMET). Additionally, we identified that SHIP2 associates with VEGFR3 following VEGFC stimulation in PLA assays (**Figure 6B**; PLA signal from interaction of FLAG and VEGFR3). Negative controls included the omission of one primary antibody during the PLA reaction (**Figure S5A–B**). The endogenous SHIP2-cMET and SHIP2-VEGFR3 interaction was confirmed by co-immunoprecipitation of TIME (**Figure S5C**). To provide insight into how T180A SHIP2 mutation results in loss of an ability of SHIP2 to inhibit AKT and ERK activation within LEC, we also examined the effect of this mutation upon physical interaction with cMET and VEGFR3. Since T180 is in close proximity to the SHIP2 SH2 domain that is known to mediate interaction with cMET, we predicted a reduced interaction with at least this RTK. However, as determined in PLA assays, the T180A mutation did not largely affect the ability of SHIP2 to bind cMET or VEGFR3 after stimulation of cells with their respective growth factors (**Figure 6E**; *p* value = 0.56 and 0.394, respectively).

In summary, although the T180A mutation was found not to largely affect PIP3 phosphatase activity (**Fig 5G**) or significantly reduce its association with cMET or VEGFR3 *in vitro* (**Fig 6E**), this mutation results in a loss-of-function LEC phenotype (**Fig 5B–D,F**), and loss-of-function with regards activation of AKT and ERK in a cellular context (**Fig 5E**). Importantly, AKT and ERK activation in T180A transfected cells was not greater than that observed in control (vector) cells in response to either growth factor. This indicates that T180A SHIP2 mutant does not function in a dominant negative capacity to regulate the activation of AKT and ERK in the respective growth factor induced signaling pathways.

## Discussion

The goal of our study was to combine investigational near-infrared fluorescence lymphatic imaging (NIRFLI) and unbiased next generation sequencing (NGS) to discover phenotype-genotype associations in a large family with varying clinical diagnoses of lymphedema and further assess whether the identified variant has lymphatic relevance. While computed tomography (CT) or magnetic resonance imaging (MRI) lymphangiography can assess lymphatic abnormalities in symptomatic patients in rare cases, the radiographic procedure is contraindicated in asymptomatic subjects owing to procedural risks. Lymphoscintigraphy is commonly used to assess deficiency in lymph transport, but entails the use of a radioisotope to produce images that have limited temporal and spatial resolution, and can fail to identify architectural and functional abnormalities [39], [40]. Recently, we used NIRFLI in investigational studies to (i) characterize normal lymphatic architecture and function in normal control subjects; (ii) characterize abnormal lymphatic architecture and function in subjects with lymphatic disorders including reduced lymphatic contractile function and lymphatic reflux presumably from valve defects; (iii) detect early lymphatic abnormalities in a breast cancer survivor asymptomatic for cancer-acquired lymphedema [26]; and (iv) identify abnormal lymphatic architecture and function in a patient with capillary-malformation arterial venous malformations (CM-AVM) (OMIM: 608354) and Parkes Weber Syndrome (OMIM: 608355) associated with an inactivating *RASA1* mutation which was similar to that in an inducible *Rasa1* knockout mouse model [13], [35]. In this latter study, we confirmed that loss of the RASA1 protein, a negative regulator of the MAPK/ERK pathway, was directly responsible for aberrant, dysregulated lymphangiogenesis. Given the past ability of NIRF to discern subtle, subclinical lymphatic phenotypes [26], [41], and in seemingly unaffected subjects herein (for example Subjects #9 and 12) who have lymphatic abnormalities (**Fig 2C, 2D** respectively); our current study further highlights the utility of NIRFLI technique as a potential diagnostic tool.

We report here a nucleus family with inherited non-syndromic lymphedema with a distinct heterozygous non-synonymous rare mutation in the *INPPL1* gene that encodes the SHIP2 phosphatase. In addition, a heterozygous damaging mutation in the *HGF* gene that encodes HGF was identified in this family. Mutations in *HGF* and its receptor *cMET* have been associated with lymphedema beforehand [28]. Interestingly, individuals in this family that harbored mutations in both *INPPL1* and *HGF* showed more severe lymphedema symptoms. Further WES analyses of their extended family identify this T180A SHIP2 mutation in 6 other subjects, 3 of whom have various diagnoses of lymphedema. This SHIP2 mutation also overlaps with a mutation of a gene within the MAPK/ERK signaling pathway, *MAP3K7*, in 5 individuals with lymphedema. However, while computationally predicted to be damaging, it remains to be biologically validated whether MAP3K7 specifically has a cellular functional role in LEC. Nonetheless, our findings indicate that SHIP2 is a regulator of lymphatic function in humans and that inherited mutations in the *INPPL1* gene may act in concert with *HGF*, and likely *MAP3K7*, mutations to exacerbate lymphatic phenotypes.

High penetrance genes associated with lymphedema, such as *VEGFR3*, exhibit the standard, rare Mendelian inheritance pattern in patients with an extreme phenotype. However, since lymphatic abnormalities most likely arise from the interplay between several genes and environmental factors as in the case of many other disorders, lymphedema likely cannot be attributed to only single, high penetrance genes. For the majority of the more common disorders such as stroke, hypertension, obesity, autism, schizophrenia, bipolar disorder, etc. a large number of genes are involved with each one contributing to the disorder. This situation may be expected in non-syndromic lymphedema, since only *VEGFC/VEGFR3* axis genes that have been identified as the sole source of the disorder, yet 64% of patients with familial history and 92% of patients with sporadic non-syndromic lymphedema do not harbor these mutations [17], [42]. Inherited heterozygous mutations in *SHIP2* together with germline heterozygous mutations in other lymphatic-regulating genes may be sufficient for the development of lymphedema. Ideally, as evidenced by the variable phenotypes in our subjects, screening of families with syndromic lymphatic dysfunction for *INPPL1* mutations could be informative and substantiate the variable penetrance that is normally observed in lymphatic disorders. However, as suggested by NIRFLI phenotyping of aberrant lymphatic architecture and function in asymptomatic subjects, variations in normal physiologic challenges such as puberty, pregnancy, immune insults, or mechanical injury could provide physiologic and/or environmental second-hits that could account for the differences in disease expressivity and age of onset in subjects.

SHIP2 dephosphorylates PI(3,4,5)P3 at the 5′position of the inositol ring and thereby is a negative regulator of the PI3K/AKT signaling pathway. To verify this important role for SHIP2 in lymphatics, we performed SHIP2 knockdown studies in LEC. These studies confirmed that SHIP2 acts as a negative regulator of HGF- and VEGFC-induced PI3K/AKT signaling in LEC and further showed an important role for SHIP2 as a negative regulator of Ras-MAPK/ERK signaling in this cell type. SHIP2 has the potential to negatively regulate Ras-MAPK/ERK signaling through either catalytic or non-catalytic mechanisms. Non-catalytic mechanisms by which SHIP2 may inhibit MAPK activation are suggested by functional studies of SHIP1, which is homologous to SHIP2. For example in B cells, SHIP1 negatively regulates MAPK through inhibition of the binding of a Grb2/SOS Ras guanine nucleotide exchange factor complex to the Shc adapter protein, which is an event that is necessary for Ras activation [43]. In addition, through physical interaction with the p62dok adapter protein, SHIP1 is thought to promote recruitment of the Ras GTPase-activating protein to membranes that inactivates Ras [44]. Additionally, over-expression of WT SHIP2 in the CHO cell line resulted in increased insulin-induced SHIP2/Shc association, reduced Shc/Grb2 association and, consequently, reduced MAPK activation, suggesting that SHIP2, like SHIP1, competes with Grb2 for Shc [45]. However, our preliminary studies show that these observations do not extend to SHIP2 in LEC as SHIP2 does not seem to interfere with the Grb2/Shc complex in LEC (**Fig S6**). With regards the catalytic regulation of MAPK by SHIP2, PIP3 could potentially lead to membrane recruitment of pleckstrin homology (PH) domain-containing proteins, other than AKT, that subsequently amplify growth factor induced activation of Ras, as reported previously for antigen receptor signaling in lymphocytes [46]. To this end, our preliminary results indicate that exogenous PIP3 is capable of activating MAPK in LEC (**Fig S7A**). To determine the extent to which PI3K and PIP3 are required for HGF- and VEGFC-induced MAPK activation in LEC, we further examined the effect of PI3K inhibition in these two pathways. PI3K inhibition substantially reduced but did not completely block HGF- and VEGFC-induced activation of MAPK (**Fig S7B**). Neither U0126 nor LY294002 affected expression of SHIP2 (not shown), which is expected since SHIP2 is upstream of PI3K/AKT and MAPK/ERK. While the exact mechanism of SHIP2 inhibition of MAPK/ERK signaling needs to be further clarified in future studies, our initial finding is consistent with the possibility that SHIP2 uses a PIP3-dependent catalytic mechanism, either directly or as a consequence of altered PI3K activity, to negatively regulate MAPK in LEC.

SHIP2 knockdown studies in LEC also showed that this phosphatase is necessary for optimal HGF- and VEGFC-induced proliferation, adhesion, migration, and tubulogenesis, which are all key processes in lymphangiogenesis. Surprisingly, we also found that knockdown of SHIP2 in LEC results in increased apoptosis, despite that PI3K/AKT signaling is recognized to promote cell survival. While the exact mechanisms responsible for this increased apoptosis remain to be studied, it is of note that sustained MAPK signaling, itself a mitogenic pathway, has paradoxically been shown to trigger cell death [47]. In this regard, the increase in MAPK signaling in SHIP2 knockdown LEC was substantially greater than the increase in PI3K/AKT signaling (**Fig 3** **and Fig S3**). Therefore, any increased survival resulting from augmented PI3K/AKT signaling may be counterbalanced and exceeded by increased apoptosis as a consequence of increased MAPK signaling. Mechanisms of ERK-mediated cell death include both intrinsic apoptosis activation characterized by mitochondrial release of cytochrome *c* and activation of caspase-9 and extrinsic apoptosis *via* activation of caspase-8 [47]. Potentially, increased apoptosis in SHIP2 knockdown cells could account for the impairment in the other LEC functional parameters that were examined. On the other hand, in other cell types, increased MAPK activation has been shown to inhibit cell proliferation and migration directly [48], [49]. Accordingly, we are investigating whether these mechanisms are at play in the LEC context.

T180A mutation is located carboxy terminal to the SH2 domain of the SHIP2 protein. Molecular modeling predictions could not be made for the T180A mutation since the entire three-dimensional structure of SHIP2 has not yet been elucidated. Nonetheless, T180A SHIP2 showed increased PIP3 cellular levels, and reduced, but not significant, levels of PIP3 phosphatase activity as WT SHIP2 *in vitro*. However, in transfected LEC, this mutation resulted in loss of an ability of SHIP2 to function as a negative regulator of AKT and MAPK. In contrast to WT SHIP2, T180A SHIP2 was completely unable to inhibit HGF- and VEGFC-induced activation of AKT and MAPK. Thus, at least with regards AKT and MAPK activation, T180A represents a complete loss-of-function SHIP2 mutation. How the T180A mutation affects the inhibitory function of SHIP2 specifically *in vivo* is unclear at present but the mutation does not appear to relate to any impaired ability of SHIP2 to interact physically with cMET or VEGFR3. Further insight into how T180A mutation impairs SHIP2 activity is likely to come from determination of the crystal structure of the full length SHIP2 protein. Additionally, while we cannot rule out what impact that endogenous SHIP2 has in the WT- and T180A overexpressing cells, we used vector-transfectants as a control to account for any experimental differences given that a stable SHIP2-null lymphatic cell line is not currently available.

Recently both homozygous and heterozygous mutations in SHIP2 have been reported in a rare skeletal disorder, opsismodysplasia (OMIM: 258480) in both unrelated and consanguineous family members [50], [51]. Furthermore, while SHIP2 has been implicated in obesity, diabetes and insulin resistance in rodents [52], [53] and in humans [45], [54], [55]; and has been suggested to have a proto-oncogenic role in human breast cancer [34], SHIP2 has not been associated with vascular disorders. However, PTEN, which similarly to SHIP2, is a negative regulator of PI3K/AKT signaling, has been implicated in lymphatic malformations specifically Proteus-like syndrome, exhibiting medium penetrance and loss-of-function phenotype [56]. Thus, it may be expected, but remains to be tested in genetic mouse models, whether SHIP2 plays a primary role in lymphangiogenesis and lymphatic vessel maintenance. To our knowledge, the only previous evidence suggesting a role of SHIP2 in lymphatic biology is a recent transcriptomic microarray study using paired punch biopsy samples of human lymphedematous tissue versus normal samples. In that study, among ∼8400 differentially expressed genes, *INPPL1* was found to have reduced expression in lymphedematous tissue samples suggesting a role for this protein in lymphatic function [57].

Previous studies performed in epithelial cells have shown that SHIP2 binds HGF-activated cMET at Y1356 in the cMET cytoplasmic tail to effect cell scattering and spreading [33]. Here we show that SHIP2 also interacts with cMET in LEC (**Fig 6A**). In addition, we show for the first time that SHIP2 interacts with VEGFR3 in LEC (**Fig 6B**). cMET is known to couple to the PI3K signaling pathway through direct physical interaction with PI3K [58]. Similarly, VEGFR3 has recently been shown to trigger the PI3K pathway through physical interaction with PI3K [59]. Altogether, these studies reveal strong similarities in the mechanisms by which cMET and VEGFR3 activate and regulate the PI3K/AKT pathway. The finding that SHIP2 physically interacts with cMET (and VEGFR3) in LEC further substantiates the notion that this SHIP2 mutation contributes to the lymphatic abnormalities in this studied family. While we found that SHIP2 did not affect the expression of cMET or VEGFR3 upon growth factor stimulation (not shown), a role for SHIP2 in cMET and VEGFR3 signaling pathways could provide an explanation for the occurrence of more serious disease in individuals with mutations in SHIP2 together with mutations in one or the other of these RTKs. Much like mutations in *HGF* and *cMET* [28], it is still unclear why the SHIP2 mutation reported herein results in a lymphatic phenotype, given the ubiquitous expression of SHIP2. One possible explanation could be the rapid physiologic lymphatic responses to external triggers during developmental and adult lymphangiogenesis [32]. Compared to the blood vasculature, the lymphatic structure may better facilitate remodeling of capillaries, which in lymphatics are thin-walled, lack tight junctions, and either lack or have incomplete basement membranes [2]. Another explanation may be the compounding effect of mutations in SHIP2, RTKs or their ligands, or in other components of PI3K/AKT and MAPK/ERK pathways, leading to abnormal LEC signal transduction and subsequently to lymphatic dysfunction.

## Supporting Information

Figure S1 **WES analysis and validation by Sanger sequencing.** (**A**) List of mutations identified in the nucleus family by whole exome sequencing (WES) and validated by Sanger sequencing showing chromosomal location, nucleotide change, zygocity and amino acid change. Check mark (✓) depicts identified mutation in corresponding subject. (**B**) Validation of WES results by Sanger sequencing showing chromatograms of T180A-SHIP2 SNP. At position of interest (highlighted in yellow), both alleles in Subject #3 contain adenosine (A; green peak) while Subject # 1, 2 and 3 have heterozygous SNPs at the same position, one containing adenosine (A; green peak) and the 2nd containing guanosine (G; black peak) resulting in ACC→GCC.
(PDF)

Figure S2 **SHIP2 is expressed in LEC.** (**A**) Confirmation of lymphatic lineage of primary HDLEC (passage 8) and TIME (passage 30) and expression of SHIP2 in HDLEC and TIME. Cells were grown to confluency and singly stained with pan-endothelial marker anti-human CD31 (top panels) and lymphatic marker anti-human podoplanin (bottom panels). Markers are depicted in orange, isotype controls are in blue, and unstained in red. Double stains were also performed (not shown). 50,000 events per experiment, N = 3. (**B**) Primary HDLEC (passage 5) were subjected to siRNA transfection for 48 hours then plated in chamber slides. LEC were allowed to adhere, fixed and permeabilized before staining with anti-human SHIP2 antibody followed by Alexa Fluor 546 antibody (green). Cells were counterstained with Alexa 488 phalloidin to detect actin (red) and nuclear stain DRAQ5. Scale bar  = 25 µm. (**C–D**) Western blot analysis of SHIP2 expression upon transfection with four different sequences of SHIP2 siRNA (#1-4 and pool, see Materials and Methods for sequence information) in HDLEC and TIME cells. Equal loading was determined by Western blotting for actin. (**E**) RT-qPCR measurement of SHIP2 mRNA levels in HDLEC and TIME pool SHIP2 siRNA transfectants normalized to GAPDH expression levels; N = 5 experiments. Control  =  untransfected cells; Mock  =  transfection reagents only; NT  =  non-targeting control siRNA.
(PDF)

Figure S3 **Dysregulated HGF- and VEGFC-induced activation of AKT and ERK1/2 in SHIP2-deficient LEC.** HDLEC (**A,C**) and TIME (**B,D**) cells were subjected to 48 hr SHIP2 siRNA and stimulated with HGF (**A,B**) or VEGFC (**C–D**) for the indicated times. Activation of AKT and ERK was determined by fluorescent double staining Western blotting of cell lysates with both phosphospecific antibodies and antibodies to total proteins. Phosphoantibodies were detected by IRDye 680 nm (red signal) and total antibodies detected by IRDye800 nm (green signal) fluorescent secondary antibodies. MFI quantification of AKT and ERK activation shown in **Fig. 3**. SHIP2 knockdown levels shown and COX IV used as loading control. Shown are representative blots from 4 independent experiments. MW = molecular weight marker.
(PDF)

Figure S4 **Phenotype of TIME cells upon SHIP2 knockdown.** (**A**) MTS cell proliferation assay of 24 hr-transfected TIME cells in response to 1% FBS over 48 hrs; N = 3. (**B**) Representative images of cell adhesion assay of siRNA-transfected TIME onto BSA, collagen and fibronectin. (**C**) Wound scratch cell migration assay of siRNA-transfected TIME cells immediately after and at 24 and 48 hrs after wounding. (**D**) 3D tube formation networks in response to growth factors imaged 24 hrs post plating. (**E**) AnnexinV apoptosis assay of siRNA-transfected TIME cells either untreated or following staurosporine treatment, quantification (N = 3) of total AnnexinV-positive cell populations and mean intensity of AnnexinV and propidium iodide. Data presented as means±SEM. *p<0.05. Scale bar  = 50 µm. Control  =  untransfected cells; NT = non-targeting siRNA.
(PDF)

Figure S5 **SHIP2 interacts with cMET and with VEGFR3.** Proximity ligation assay (PLA) negative controls of SHIP2 interaction and immunoprecipitation depicting endogenous SHIP2 interacting with cMET and VEGFR3 (**A**) SHIP2FLAG-CMET and **(B**) SHIP2FLAG-VEGFR3 interaction in TIME cells following (**A**) HGF stimulation and (**B**) VEGFC stimulation, whereby a single primary antibody is used, the second primary antibody is omitted and both PLA probes added. Note lack of red PLA signal. Blue  =  DAPI, green  =  actin and red  =  PLA fluorescence with each red dot depicting *in situ* interaction site. Scale bar  = 50 µm, bottom right panel. (**C**) Confluent naïve TIME cells were serum-starved overnight and subjected to HGF and VEGFC stimulation for 15 minutes before harvesting and lysates subjected to immunoprecipitation with anti-cMET and anti-VEGFR3 antibodies pre-absorbed with agarose beads. Immunocomplexes were subjected to immunoblotting, blots probed with anti-SHIP2 antibody, and GAPDH used to determine equal loading in whole cell lysates pre-IP.
(PDF)

Figure S6 **SHIP2 does not seem to interfere with Shc/Grb2 complex in LEC.** TIME cells were transfected with WT SHIP2 (overexp SHIP2) or empty vector for 48 hours, serum-starved for 16 hours followed by HGF or VEGFC stimulation. Total lysates were harvested and subjected to Shc immunoprecipitation using anti-Shc antibody pre-absorbed with agarose beads. Immunocomplexes were probed for SHIP2, phosphoTyrosine (pTyr99), Grb2 and Shc using standard fluorescent immunoblotting techniques. The amount of SHIP2 associated with Shc was not increased over the amount of Grb2 associated with Shc.
(PDF)

Figure S7 **PIP3 and PI3K influence MAPK activation in LEC.** (**A**) Increasing concentrations of recombinant PI(3,4,5)P3 was delivered into TIME cells using Shuttle PIP kit for 1 hour and cells subsequently lysed and subjected to immunoblotting against pAKT and pERK1/2 antibodies. (**B**) PI3K inhibition reduces HGF- and VEGFC-induced ERK activation in LEC. TIME cells were treated with LY294002 (PI3K inhibitor) and U0126 (MEK inhibitor) prior to 5 min growth-factor stimulation. Activation of ERK was determined by Western blotting of cell lysates with double staining of pERK1/2 antibody (red signal) and total ERK1/2 antibody (green signal) to demonstrate equal loading. MW  =  molecular weight marker.
(PDF)

Video S1 **Video illustrating the normal lymphatic contractile function in the right foot of a 43 year old male control subject.** The bandages near the bottom of the video cover the injection sites on the top of the foot.
(AVI)

Video S2 **Video illustrating the reduced lymphatic contractile function in the right “unaffected” foot of Subject# 1 (left unilateral** ***praecox*** **LE) which shows lymphatic capillaries that radiate from the injection site and with some exhibiting abnormal retrograde flow towards the toes.** The round and square bandages cover the injection sites on the top of the foot.
(AVI)

Video S3 **Video illustrating the normal lymphatic contractile function in the right shin of a 43 year old male control subject.** The bright spots on the left and right of the calf are the injection sites.
(AVI)

Video S4 **Video illustrating the altered lymphatic contractility in the right “unaffected” shin of Subject #1 (left unilateral praecox LE) showing a vessel crossing over the shin just below the knee.**
(AVI)
