# Supplementary material for: Evidence for SH2 Domain-Containing 5′-Inositol Phosphatase-2 (SHIP2) Contributing to a Lymphatic Dysfunction
Source: PLoS One. 2014 Nov 10;9(11):e112548. doi: 10.1371/journal.pone.0112548 (PMC4226566; doi:10.1371/journal.pone.0112548)

Supplementary Figure 1

A

Nucleus Family									
**Gene**	**Subject #1**	**Subject #2**	**Subject #3**	**Subject #4**	**Chromosome**	**Location (Hg19)**	**Nucleotide change**	**Zygocity**	**Amino acid change**
INPPL1	✓	✓		✓	chr11	71940153	A > G	Heterozygote	T180A
HGF	✓	✓	✓		chr7	81359017	C > A	Heterozygote	G315V

B

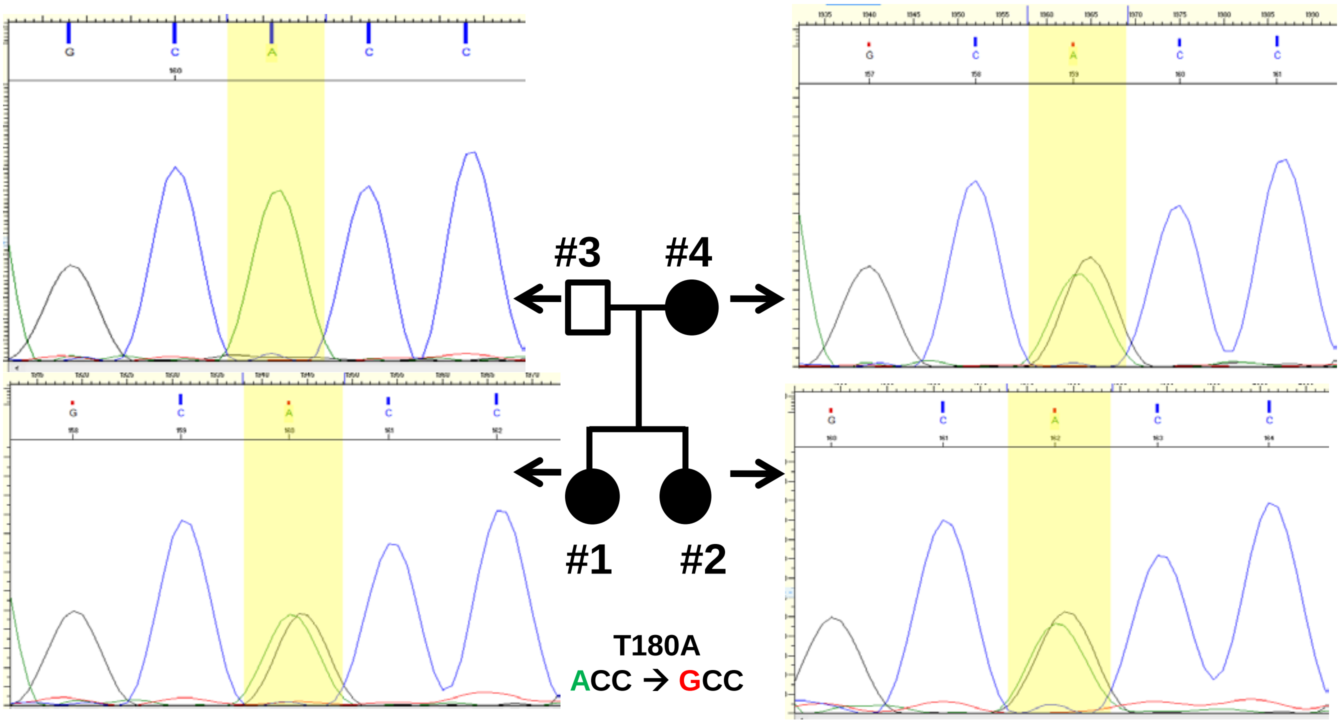

Supplement: Figure S1 — WES analysis and validation by Sanger sequencing. (A) List of mutations identified in the nucleus family by whole exome sequencing (WES) and validated by Sanger sequencing showing chromosomal location, nucleotide change, zygocity and amino acid change. Check mark (✓) depicts identified mutation in corresponding subject. (B) Validation of WES results by Sanger sequencing showing chromatograms of T180A-SHIP2 SNP. At position of interest (highlighted in yellow), both alleles in Subject #3 contain adenosine (A; green peak) while Subject # 1, 2 and 3 have heterozygous SNPs at the same position, one containing adenosine (A; green peak) and the 2nd containing guanosine (G; black peak) resulting in ACC→GCC. (PDF) [file pone.0112548.s001.pdf]
