# Supplementary figures and images for: Evidence for SH2 Domain-Containing 5′-Inositol Phosphatase-2 (SHIP2) Contributing to a Lymphatic Dysfunction
Source: PLoS One. 2014 Nov 10;9(11):e112548. doi: 10.1371/journal.pone.0112548 (PMC4226566; doi:10.1371/journal.pone.0112548)

Supplementary Figure 2

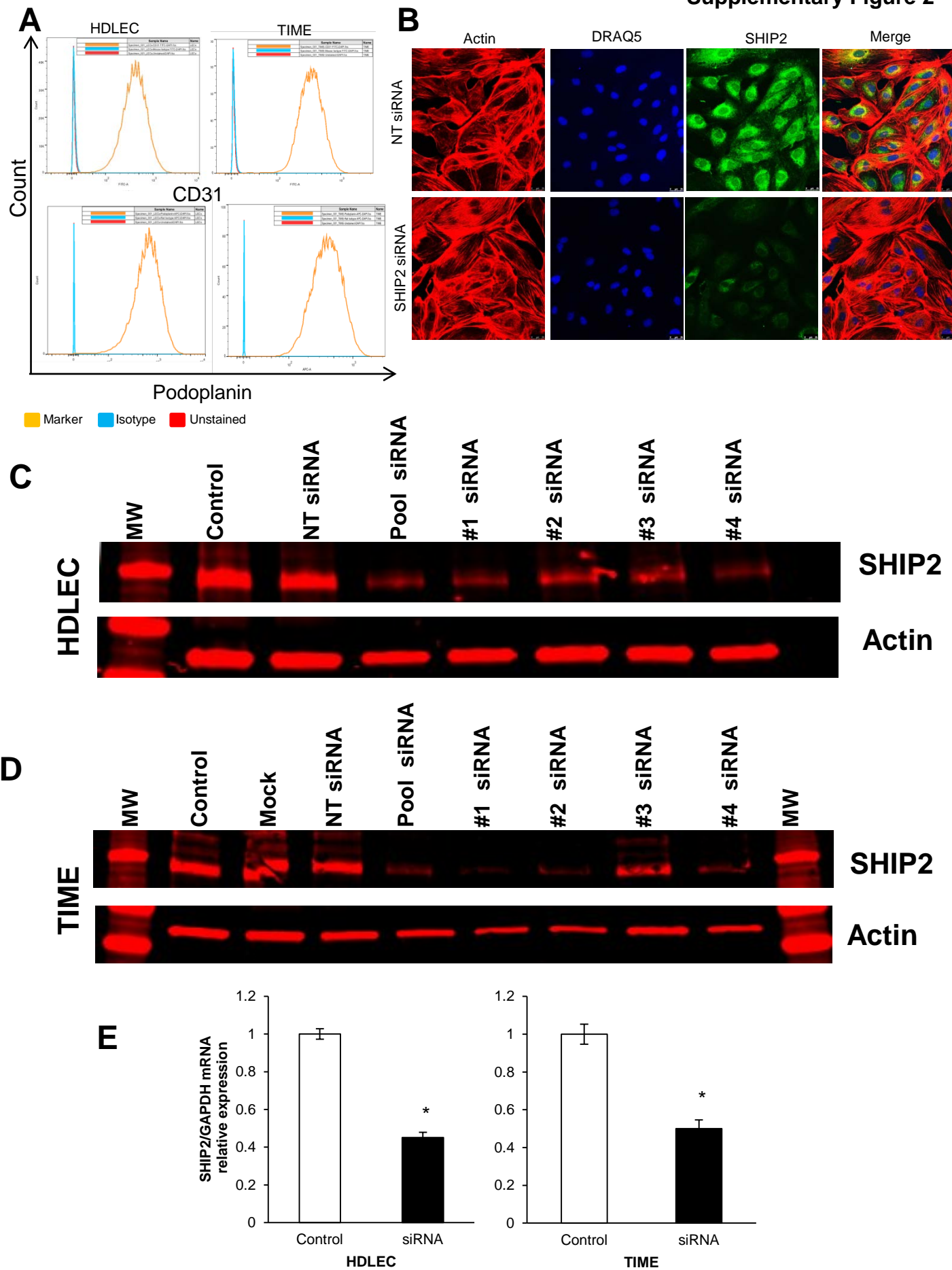

Supplement: Figure S2 — SHIP2 is expressed in LEC. (A) Confirmation of lymphatic lineage of primary HDLEC (passage 8) and TIME (passage 30) and expression of SHIP2 in HDLEC and TIME. Cells were grown to confluency and singly stained with pan-endothelial marker anti-human CD31 (top panels) and lymphatic marker anti-human podoplanin (bottom panels). Markers are depicted in orange, isotype controls are in blue, and unstained in red. Double stains were also performed (not shown). 50,000 events per experiment, N = 3. (B) Primary HDLEC (passage 5) were subjected to siRNA transfection for 48 hours then plated in chamber slides. LEC were allowed to adhere, fixed and permeabilized before staining with anti-human SHIP2 antibody followed by Alexa Fluor 546 antibody (green). Cells were counterstained with Alexa 488 phalloidin to detect actin (red) and nuclear stain DRAQ5. Scale bar = 25 µm. (C–D) Western blot analysis of SHIP2 expression upon transfection with four different sequences of SHIP2 siRNA (#1-4 and pool, see Materials and Methods for sequence information) in HDLEC and TIME cells. Equal loading was determined by Western blotting for actin. (E) RT-qPCR measurement of SHIP2 mRNA levels in HDLEC and TIME pool SHIP2 siRNA transfectants normalized to GAPDH expression levels; N = 5 experiments. Control = untransfected cells; Mock = transfection reagents only; NT = non-targeting control siRNA. (PDF) [file pone.0112548.s002.pdf]

## HGF

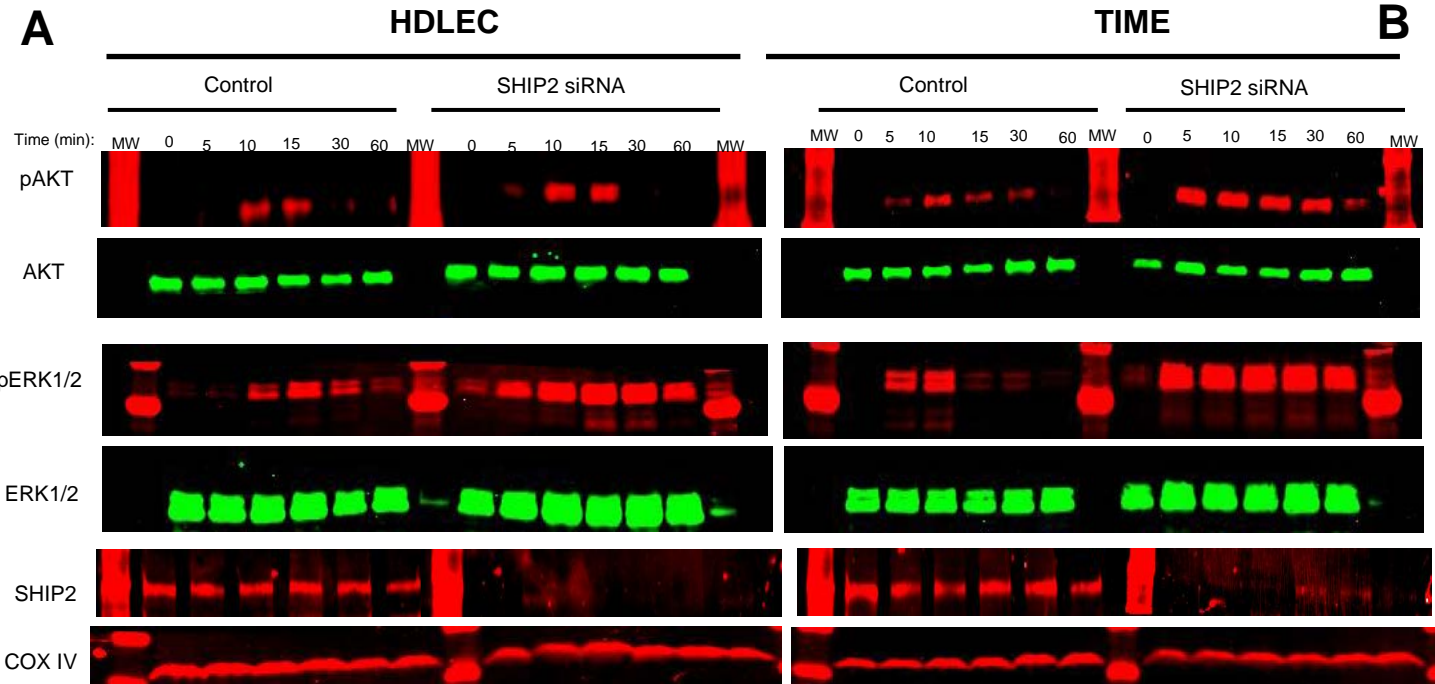

## VEGFC

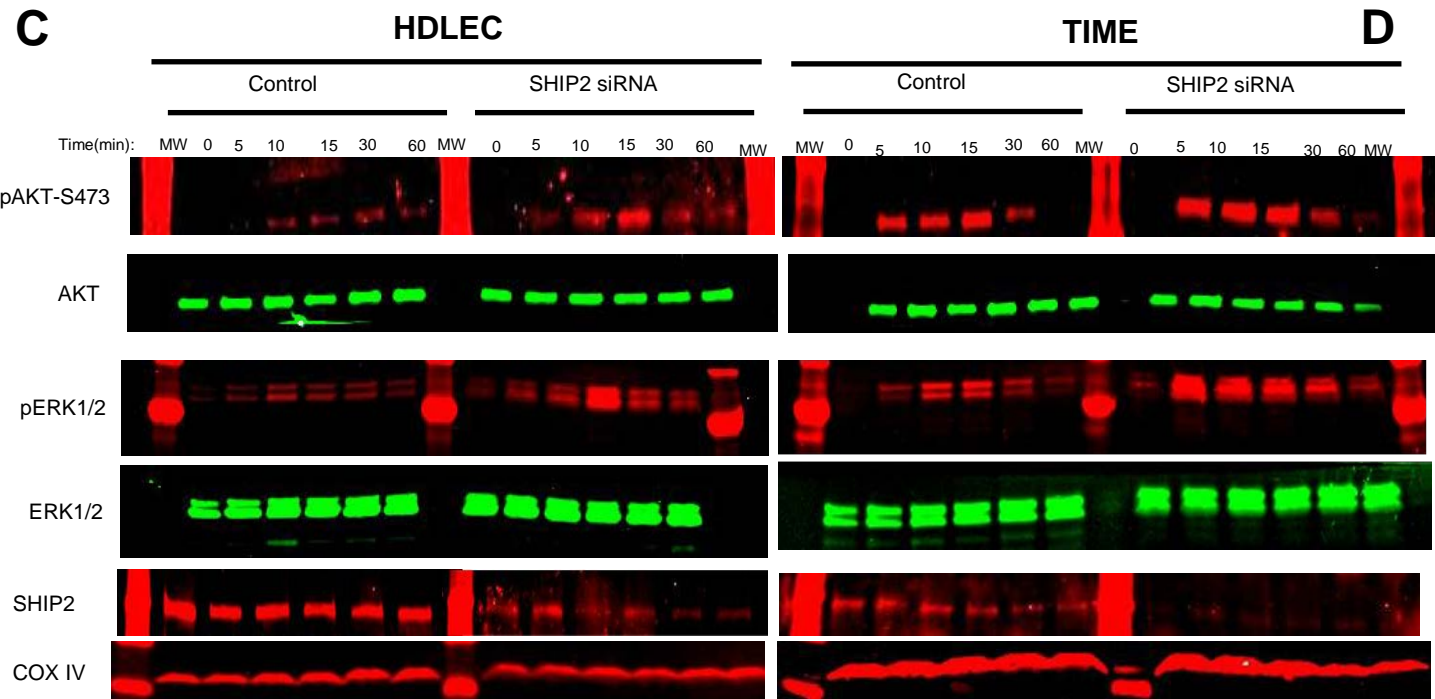

Supplement: Figure S3 — Dysregulated HGF- and VEGFC-induced activation of AKT and ERK1/2 in SHIP2-deficient LEC. HDLEC (A,C) and TIME (B,D) cells were subjected to 48 hr SHIP2 siRNA and stimulated with HGF (A,B) or VEGFC (C–D) for the indicated times. Activation of AKT and ERK was determined by fluorescent double staining Western blotting of cell lysates with both phosphospecific antibodies and antibodies to total proteins. Phosphoantibodies were detected by IRDye 680 nm (red signal) and total antibodies detected by IRDye800 nm (green signal) fluorescent secondary antibodies. MFI quantification of AKT and ERK activation shown in Fig. 3 . SHIP2 knockdown levels shown and COX IV used as loading control. Shown are representative blots from 4 independent experiments. MW = molecular weight marker. (PDF) [file pone.0112548.s003.pdf]

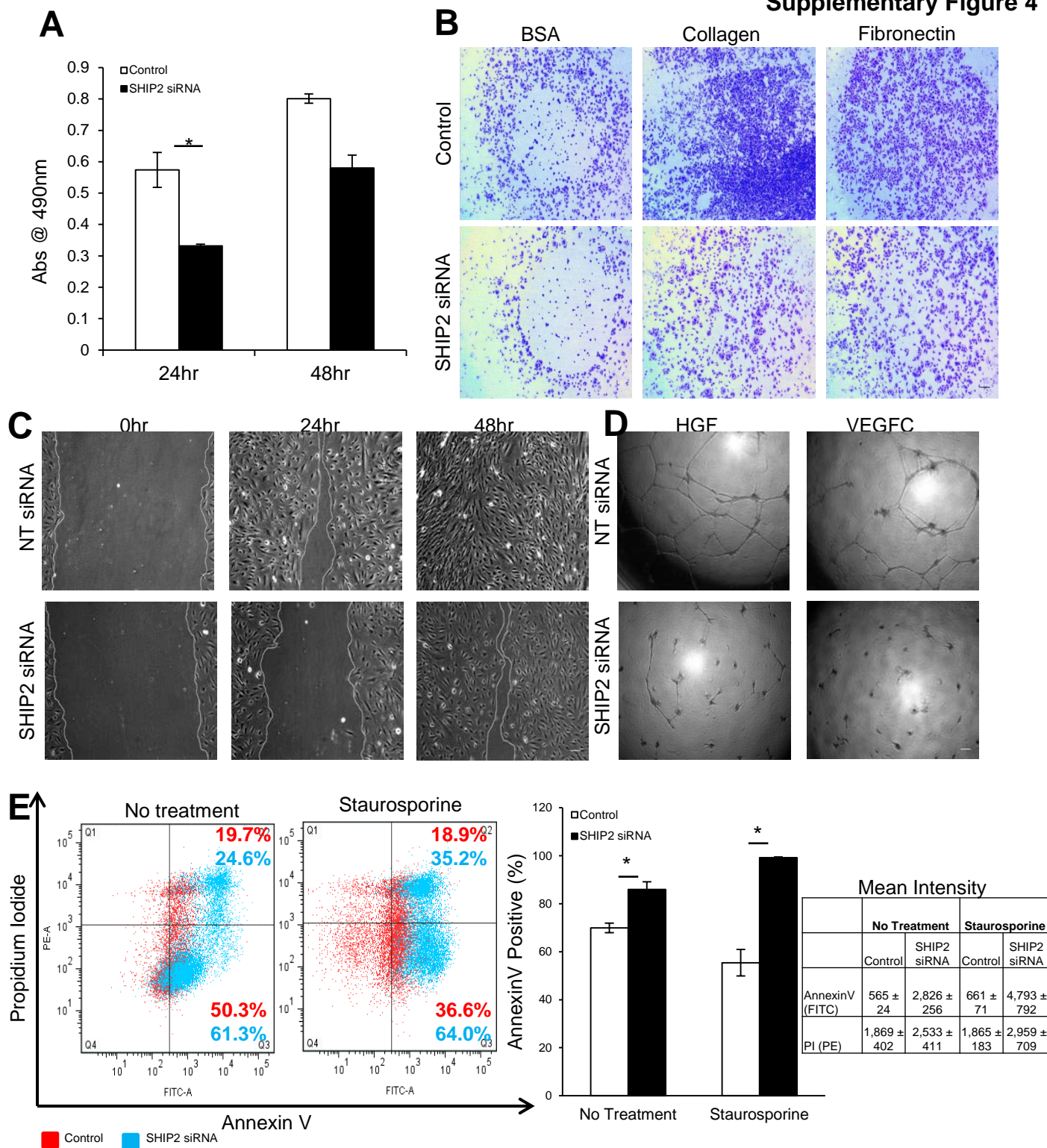

Supplement: Figure S4 — Phenotype of TIME cells upon SHIP2 knockdown. (A) MTS cell proliferation assay of 24 hr-transfected TIME cells in response to 1% FBS over 48 hrs; N = 3. (B) Representative images of cell adhesion assay of siRNA-transfected TIME onto BSA, collagen and fibronectin. (C) Wound scratch cell migration assay of siRNA-transfected TIME cells immediately after and at 24 and 48 hrs after wounding. (D) 3D tube formation networks in response to growth factors imaged 24 hrs post plating. (E) AnnexinV apoptosis assay of siRNA-transfected TIME cells either untreated or following staurosporine treatment, quantification (N = 3) of total AnnexinV-positive cell populations and mean intensity of AnnexinV and propidium iodide. Data presented as means±SEM. *p<0.05. Scale bar = 50 µm. Control = untransfected cells; NT = non-targeting siRNA. (PDF) [file pone.0112548.s004.pdf]

**Supplementary Figure 6**

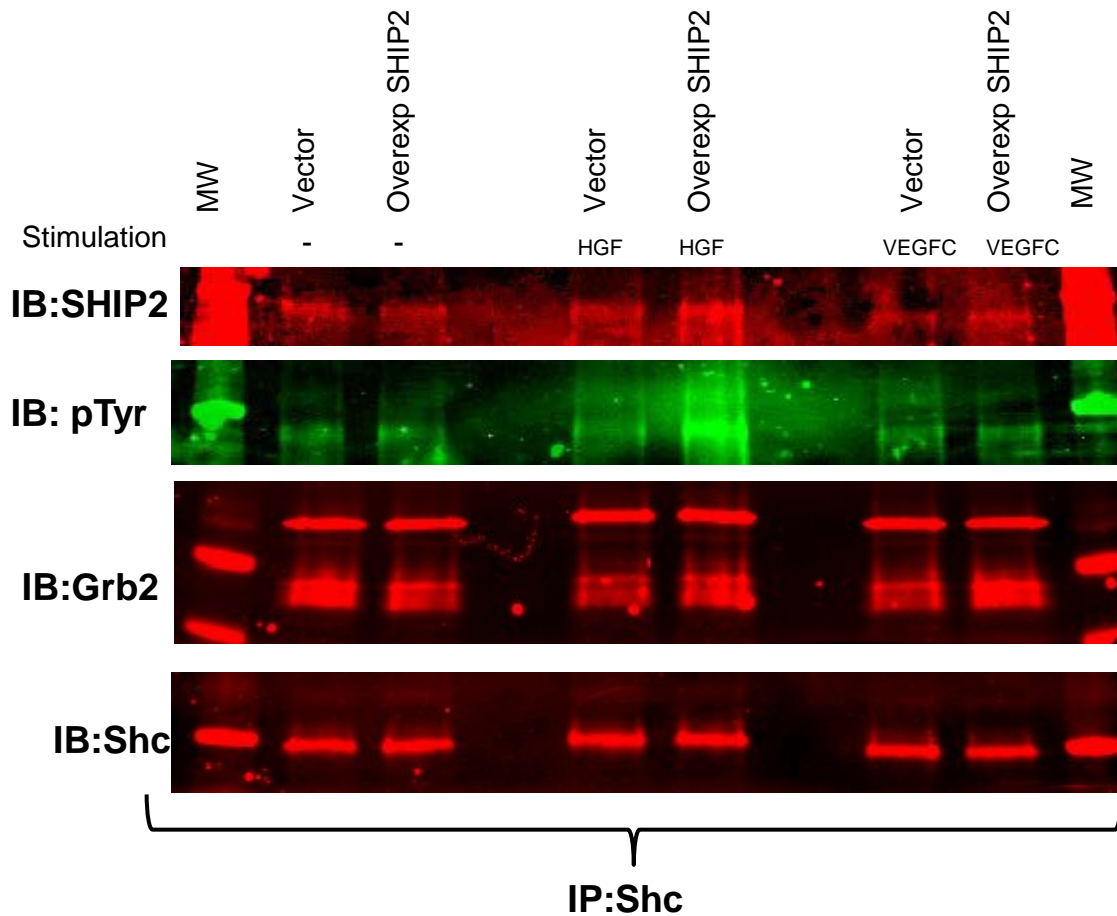

Supplement: Figure S6 — SHIP2 does not seem to interfere with Shc/Grb2 complex in LEC. TIME cells were transfected with WT SHIP2 (overexp SHIP2) or empty vector for 48 hours, serum-starved for 16 hours followed by HGF or VEGFC stimulation. Total lysates were harvested and subjected to Shc immunoprecipitation using anti-Shc antibody pre-absorbed with agarose beads. Immunocomplexes were probed for SHIP2, phosphoTyrosine (pTyr99), Grb2 and Shc using standard fluorescent immunoblotting techniques. The amount of SHIP2 associated with Shc was not increased over the amount of Grb2 associated with Shc. (PDF) [file pone.0112548.s006.pdf]

**A**

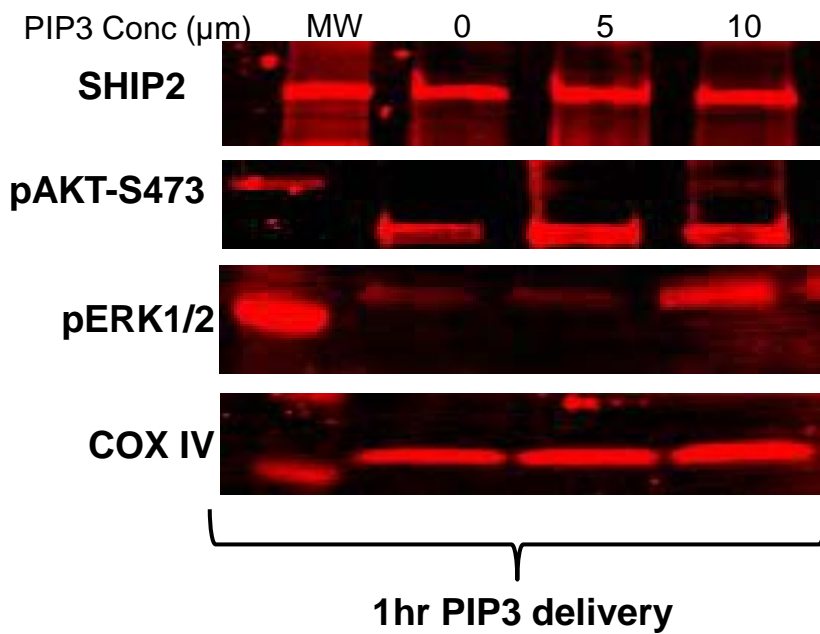

**B**

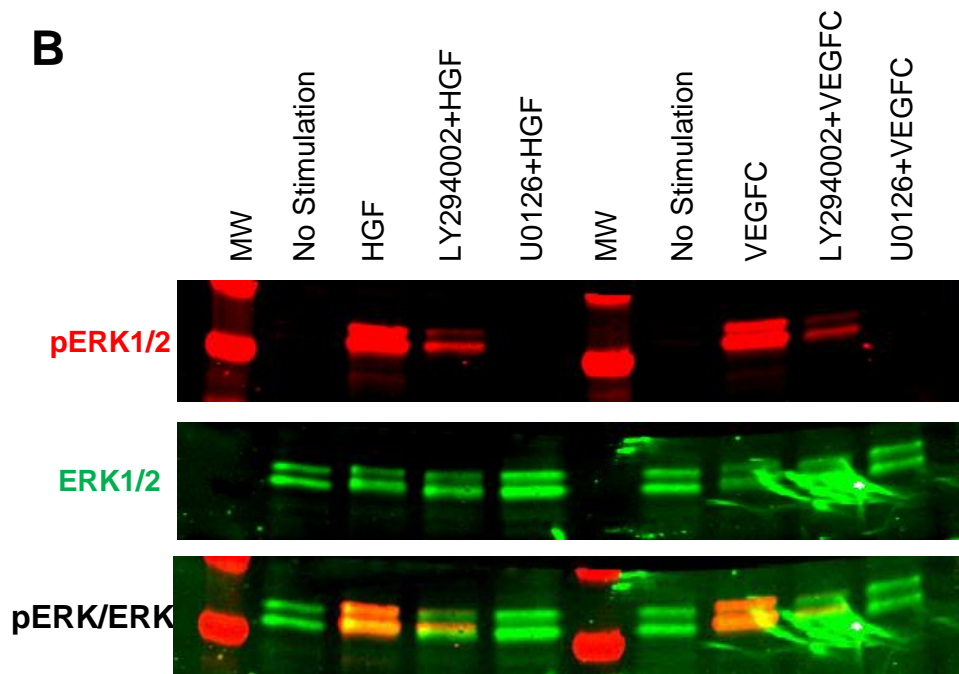

Supplement: Figure S7 — PIP3 and PI3K influence MAPK activation in LEC. (A) Increasing concentrations of recombinant PI(3,4,5)P3 was delivered into TIME cells using Shuttle PIP kit for 1 hour and cells subsequently lysed and subjected to immunoblotting against pAKT and pERK1/2 antibodies. (B) PI3K inhibition reduces HGF- and VEGFC-induced ERK activation in LEC. TIME cells were treated with LY294002 (PI3K inhibitor) and U0126 (MEK inhibitor) prior to 5 min growth-factor stimulation. Activation of ERK was determined by Western blotting of cell lysates with double staining of pERK1/2 antibody (red signal) and total ERK1/2 antibody (green signal) to demonstrate equal loading. MW = molecular weight marker. (PDF) [file pone.0112548.s007.pdf]
