# Supplementary material for: Evidence for SH2 Domain-Containing 5′-Inositol Phosphatase-2 (SHIP2) Contributing to a Lymphatic Dysfunction
Source: PLoS One. 2014 Nov 10;9(11):e112548. doi: 10.1371/journal.pone.0112548 (PMC4226566; doi:10.1371/journal.pone.0112548)

Supplementary Figure 5

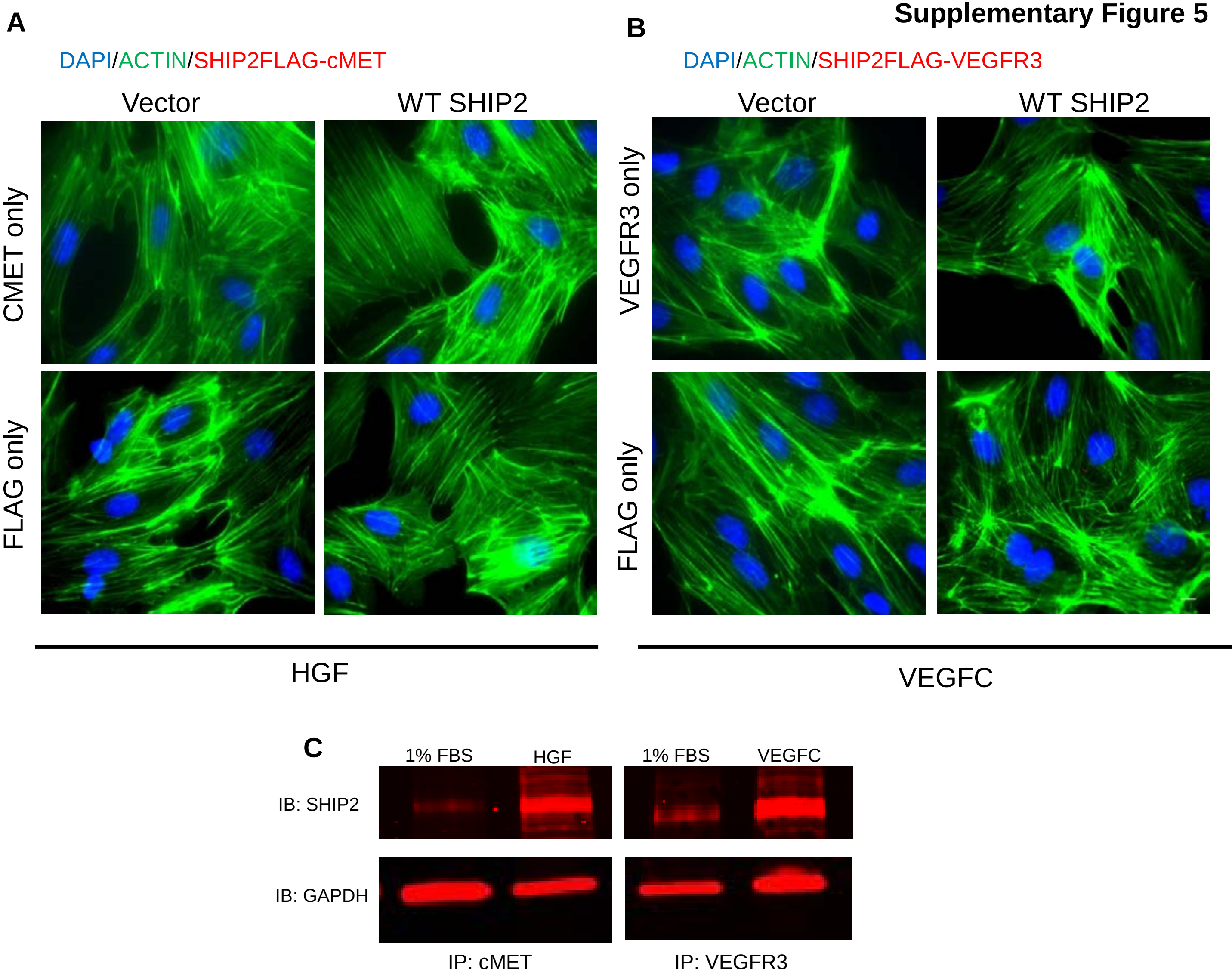

Supplement: Figure S5 — SHIP2 interacts with cMET and with VEGFR3. Proximity ligation assay (PLA) negative controls of SHIP2 interaction and immunoprecipitation depicting endogenous SHIP2 interacting with cMET and VEGFR3 (A) SHIP2FLAG-CMET and (B) SHIP2FLAG-VEGFR3 interaction in TIME cells following (A) HGF stimulation and (B) VEGFC stimulation, whereby a single primary antibody is used, the second primary antibody is omitted and both PLA probes added. Note lack of red PLA signal. Blue = DAPI, green = actin and red = PLA fluorescence with each red dot depicting in situ interaction site. Scale bar = 50 µm, bottom right panel. (C) Confluent naïve TIME cells were serum-starved overnight and subjected to HGF and VEGFC stimulation for 15 minutes before harvesting and lysates subjected to immunoprecipitation with anti-cMET and anti-VEGFR3 antibodies pre-absorbed with agarose beads. Immunocomplexes were subjected to immunoblotting, blots probed with anti-SHIP2 antibody, and GAPDH used to determine equal loading in whole cell lysates pre-IP. (PDF) [file pone.0112548.s005.pdf]
